# Efficacy and safety of baricitinib in hospitalized adults with severe or critical COVID-19 (Bari-SolidAct): a randomised, double-blind, placebo-controlled phase 3 trial

**DOI:** 10.1186/s13054-022-04205-8

**Published:** 2023-01-10

**Authors:** Marius Trøseid, José R. Arribas, Lambert Assoumou, Aleksander Rygh Holten, Julien Poissy, Vida Terzić, Fulvia Mazzaferri, Jesús Rodríguez Baño, Joe Eustace, Maya Hites, Michael Joannidis, José-Artur Paiva, Jean Reuter, Isabel Püntmann, Thale D. J. H. Patrick-Brown, Elin Westerheim, Katerina Nezvalova-Henriksen, Lydie Beniguel, Tuva Børresdatter Dahl, Maude Bouscambert, Monika Halanova, Zoltán Péterfi, Sotirios Tsiodras, Michael Rezek, Matthias Briel, Serhat Ünal, Martin Schlegel, Florence Ader, Karine Lacombe, Cecilie Delphin Amdal, Serge Rodrigues, Kristian Tonby, Alexandre Gaudet, Lars Heggelund, Joy Mootien, Asgeir Johannessen, Jannicke Horjen Møller, Beatriz Diaz Pollan, Anders Aune Tveita, Anders Benjamin Kildal, Jean-Christophe Richard, Olav Dalgard, Victoria Charlotte Simensen, Aliou Baldé, Lucie de Gastines, Marta del Álamo, Burç Aydin, Fridtjof Lund-Johansen, Mary-Anne Trabaud, Alpha Diallo, Bente Halvorsen, John-Arne Røttingen, Evelina Tacconelli, Yazdan Yazdanpanah, Inge C. Olsen, Dominique Costagliola, EU SolidAct study group, Anne Ma Dyrhol-Riise, Birgitte Stiksrud, Synne Jenum, Magnhild Eide MacPherson, Nikolai Ravn Aarskog, Kjerstin Røstad, Linda Gail Skeie, Åsne Dahl, Jeanette Konstance Steen, Sarah Nur, Filip Segers, Katrine Andersen Korsan, Ashwini Sethupathy, Ann Jorunn Sandstå, Gunn-Janne Paulsen, Thor Ueland, Annika Michelsen, Pål Aukrust, Jan Erik Berdal, Ingunn Melkeraaen, Merete Moen Tollefsen, Jessica Andreassen, Jannicke Dokken, Karl Erik Müller, Bjørn Martin Woll, Hanne Opsand, Mette Bogen, Linn-Therese Rød, Trude Steinsvik, Bjørn Åsheim-Hansen, Randi Haukaas Bjerkreim, Åse Berg, Solfrid Moen, Stina Kvalheim, Kristian Strand, Berit Gravrok, Vegard Skogen, Elias Myrvoll Lorentzen, Simen Walberg Schive, Lasse Rossvoll, Hedda Hoel, Simon Engebråten, Mia Schie Martinsson, Monica Thallinger, Elise Ådnanes, Raisa Hannula, Nina Bremnes, Kristin Liyanarachi, Birgitta Ehrnström, Martin Kvalshaug, Kari Berge, Marte Bygdås, Linda Gustafsson, Saad AballiB, Marianne Strand, Britt Andersen, Pål Aukrust, Andreas Barratt-Due, Katerina Nezvalova Henriksen, Trine Kåsine, Anne Ma Dyrhol-Riise, Jan Erik Berdal, Raphaël Favory, Saad Nseir, Sebastien Preau, Mercé Jourdain, Geoffrey Ledoux, Arthur Durand, Marion Houard, Anne-Sophie Moreau, Anahita Rouzé, Romain Tortuyaux, Guillaume Degouy, Clémentin Levy, Vincent Liu, Nicolas Dognon, Laure Mariller, Claire Delcourte, Zineb Reguig, Amélie Cerf, Marie Cuvelliez, Eric Kipnis, Marielle Boyer-Beysserre, Anne Bignon, Laurie Parmentier, Damia Meddour, Sarah Frade, Jean-François Timsit, Nathan Peiffer-Smadja, Paul-Henri Wicky, Etienne De Montmollin, Lila Bouadma, Julien Dessajan, Romain Sonneville, Juliette Patrier, Simona Presente, Zmihi Sylia, Christophe Rioux, Michaël Thy, Lio Collias, Yasmine Bouaraba, Nikita Dobremel, Anne-Florence Dureau, Pierre Oudeville, Valentin Pointurier, Yannick Rabouel, Laure Stiel, Camille Alzina, Camille Ramstein, Hafid Ait-Oufella, Fatima Hamoudi, Thomas Urbina, Yoann Zerbib, Julien Maizel, Celine Wilpotte, Lionel Piroth, Mathieu Blot, Thibault Sixt, Florian Moretto, Carole Charles, Sandrine Gohier, Damien Roux, Camille Le Breton, Coralie Gernez, Ingrid Thiry, Loredana Baboi, Denis Malvy, Alexandre Boyer, Pauline Perreau, Maddalena Armellini, Giulia De Luca, Ospedale S. M. Massimo Di Pietro, Benedetta Romanin, Michela Brogi, Francesco Castelli, Silvia Amadasi, Francesco Barchiesi, Benedetta Canovari, Nicola Coppola, Mariantonietta Pisaturo, Antonio Russo, Laura Occhiello, Francesco Cataldo, Marta Mora Rillo, Javier Queiruga, Enrique Seco, Stefan Stewart, Alberto M. Borobia, Paloma Moraga, Rocío Prieto, Irene García, Carlota Rivera, José Luis Narro, Natalia Chacón, Sandra de la Rosa, María Macías, Lydia Barrera, Almudena Serna, Virginia Palomo, Maria Isabel García Sánchez, David Gutiérrez, Ana Silva Campos, Miguel Ángel Gómez Garfia, Elvira Bonilla Toyos, Judith Sanabria Cabrera, María Isabel Lucena, Eva Larranaga Lapique, Pierre Englert, Zineb Khalil, Frédérique Jacobs, Justine Malaise, Odette Mukangenzi, Cinderella Smissaert, Marc Hildebrand, Delphine Martiny, Audrey Vervacke, Axelle Scarnière, Nicolas Yin, Charlotte Michel, Lucie Seyler, Sabine Allard, Johan Van Laethem, Gil Verschelden, Annelies Meeuwissen, Alex De Waele, Virgini Van Buggenhout, Dora Monteyne, Nils Noppe, Leila Belkhir, Jean Cyr Yombi, Julien De Greef, Jean Baptiste Mesland, Léopold De Ghellinck, Valérie Kin, Céline D’Aoust, Anne Bouvier, Anne- Charlotte Dekeister, Estelle Hawia, Adeline Gaillet, Hélène Deshorme, Severine Halleux, Vanessa Galand, Roberto Roncon-Albuquerque, Luís Linhares Santos, César Burgi Vieira, Rosana Magalhaes, Sónia Ferreira, Mariana Bernardo, Arthur Jackson, Corinna Sadlier, Sarah O’Connell, Matthew Blair, Edmund Manning, Fiona Cusack, Niamh Kelly, Hannah Stephenson, Ruben Keane, Aisling Murphy, Michele Cunnane, Fionnuala Keane, Mary-Claire O’Regan, Eoghan de Barra, Aimee McGreal Bellone, Siobhan O’Regan, Patrick Carey, Jeffrey Harte, Peter Coakley, Aoife Heeney, Dorothy Ryan, Gerard Curley, Samuel McConkey, Imran Sulaiman, Richard Costello, Cora McNally, Claire Foley, Sophie Trainor, Benson Jacob, Suchitra Vengathodi, Brian Kent, Colm Bergin, Liam Townsend, Colm Kerr, Nalini Panti, Alberto Garcia Sanz, Binny Benny, Edel O. Dea, Niamh Galvin, Claire Burke, Aisling Galvin, Sara Aisiyabi, Deepanjali Lobo, John Laffey, Bairbre McNicolas, David Cosgrave, J. R. Sheehan, Ciprian Nita, Ciara Hanley, Claire Kelly, Maeve Kernan, Jonathan Murray, Thérèse Staub, Thomas Henin, Gaelle Damilot, Tania Bintener, Joelle Colling, Christian Ferretti, Christophe Werer, Pascal Stammet, Pierre Braquet, Vic Arendt, Esther Calvo, Christian Michaux, Chouaib Mediouni, Ali Znati, Gloria Montanes, Laetitia Garcia, Claudius Thomé, Robert Breitkopf, Andreas Peer, Georg Lehner, Romuald Bellman, Adelheid Ditlbacher, Armin Finkenstedt, Klemens Zotter, Christian Preuss Hernandez, Sasa Rajsic, Barbara Lanthaler, Richard Greil, Kiss Tamás, Szilvia Kovácsné-Levang, David Sipos, Agnes Kappéter, Bernadett Halda-Kiss, Edit Madarassi-Papp, Edit Hajdu, Balázs Bende, Thomas Konstantinos, Charalambos Moschopoulos, Eleni Labrou, Maria Tsakona, Ioannis Grigoropoulos, Anastasia Kotanidou, Paraskevi Fragkou, Maria Theodorakopoulou, Eugenia Pantazi, Edison Jahai, Maria Moukouli, Dimitrios Siafakas, Bernd Mühlbauer, Rolf Dembinski, Kathrin Stich, Gerhard Schneider, Andrej Nagy, Karolína Grodová, Michaela Kubelová, Lenka Součková, Helena Kartáková Švábová, Regina Demlová, Simona Sonderlichová, Serhat Unal, A. C. Inkaya, Stephanie de Bono, Cynthia E. Kartman, David H. Adams, Brenda Crowe, Yazdan Yazdanapanah, Serhat Unal, Gerhard Schneider, Bernd Mühlbauer, Tone Ødegård, Gine Bakkehøi, Brigitte Autran, Magnar Bjørås, Xavier de- Lambellerie, Fulvia Mezzarri, Jeremie Guedj, Helene Esperou, Julia Lumbroso, Tobias Welte, Alexandra Calmy, Søren Pischke, Shaun Treweek, Els Goetghebeur, Adelaide Doussau, Laurence Weiss, Frank Hulstaert, Radu Botgros, Marta del Alamo, Florence Chung, Julia Lumbroso, Markus Zeitlinger, Begonya N. Escalera, Chantal Csajka, Clare Williams, Alain Amstutz, Corina Silvia Rüegg, Charles Burdet, Clement Massonnaud, Drifa Belhadi, France Mentré, Massinissa Aroun, France Mentré, Stephan Ehrmann, Helene Espoerou, Charles Burdet, Ragnhild Sørum Falk, Kristin Bjordal, Gina Bakkehøi, Tone Ødegård, Andreas Barratt-Due

**Affiliations:** 1 Section for Clinical Immunology and Infectious Diseases, Oslo University Hospital, Oslo, Norway; 2 Institute of Clinical Medicine, University of Oslo, Oslo, Norway; 3 Infectious Diseases Unit, Internal Medicine Department, La Paz University Hospital, IdiPAZ, Madrid, Spain; 4 Centro de Investigación Biomédica en Red de Enfermedades Infecciosas (CIBERINFEC), Madrid, Spain; 5 Sorbonne Université, INSERM, Institut Pierre Louis d’Épidémiologie Et de Santé Publique (IPLESP), Paris, France; 6 Department of Acute Medicine, Oslo University Hospital, Oslo, Norway; 7 Lille University, Lille, France/CHU Lille - Hôpital Roger Salengro, Lille, France; 8 L’Institut National de La Santé Et de La Recherche Médicale (Inserm), Paris, France; 9 Maladies Infectieuses Emergentes, 75015 Paris, France; 10 Institut National de La Santé Et de La Recherche Médicale, INSERM, 75013 Paris, France; 11 Division of Infectious Diseases, Department of Diagnostics and Public Health, University of Verona, Verona, Italy; 12 Department of Medicine, Virgen Macarena University Hospital, Seville, Spain; 13 University of Sevilla and Biomedicines Institute of Seville (IBiS)/CSIC, Seville, Spain; 14 CIBERINFEC, Instituto de Salud Carlos III, Madrid, Spain; 15 University College Cork, Cork, Ireland; 16 Brussels University Hospital-Erasme, Brussels, Belgium; 17 Université Libre de Bruxelles, Brussels, Belgium; 18 Medical University Innsbruck, Innsbruck, Austria; 19 Intensive Care Medicine Department, Centro Hospitalar Universitário Sao Joao, Porto, Portugal; 20 Faculty of Medicine, University of Porto, Porto, Portugal; 21 Centre Hospitalier de Luxembourg, Service de Réanimation-Soins Intensifs, 1210 Luxembourg, Luxembourg; 22 Institute of Pharmacology, Hospital Group Gesundheit Nord gGmbH, Bremen, Germany; 23 Division of Surgery, Inflammatory Diseases and Transplantation, Oslo University Hospital, Oslo, Norway; 24 Section for Monitoring, Clinical Trial Unit (CTU), Oslo University Hospital, Oslo, Norway; 25 Department of Haematology, Oslo University Hospital and Oslo Hospital Pharmacy, Oslo, Norway; 26 Research Institute for Internal Medicine, Oslo University Hospital, Oslo, Norway; 27 Division of Emergencies and Critical Care, Oslo University Hospital, Oslo, Norway; 28 Laboratoire de Virologie, Institut Des Agents Infectieux de Lyon, Centre National de Reference Des Virus Des Infections Respiratoires France Sud, Hospices Civils de Lyon, 69317 Lyon, France; 29 Department of Epidemiology, Faculty of Medicine, Pavol Jozef Šafárik University in Košice, Košice, Slovakia; 30 1St Department of Internal Medicine, Division of Infectology, University of Pécs, Pécs, Hungary; 31 National and Kapodistrian University of Athens, Athens, Greece; 32 University Hospital of Athens Attikon, Athens, Greece; 33 St. Anne University Hospital, Brno, Czech Republic; 34 Swiss Clinical Trial Organisation and Department of Clinical Research, University Hospital Basel and University of Basel, Basel, Switzerland; 35 Hacettepe University Hospital, Ankara, Turkey; 36 Department of Anesthesiology and Intensive Care Medicine, Klinikum Rechts Der Isar, Technische Universität München, Munich, Germany; 37 Hospices Civils de Lyon, Département Des Maladies Infectieuses Et Tropicales, 69004 Lyon, France; 38 Centre International de Recherche en Infectiologie (CIRI), Inserm 1111, Université Claude Bernard Lyon 1, CNRS, UMR5308, École Normale Supérieure de Lyon, Univ Lyon, 69007 Lyon, France; 39 Sorbonne Université, Institut Pierre-Louis d’Épidemiologie Et de Santé Publique, INSERM, 75013 Paris, France; 40 APHP, Hôpital Saint-Antoine, Service de Maladies Infectieuses Et Tropicales, 75012 Paris, France; 41 Research support service and Department of Oncology, Oslo University Hospital, Oslo, Norway; 42 Deptartment of Infectious Diseases, Oslo University Hospital, Oslo, Norway; 43 Critical Care Center, Department of Intensive Care Medicine, CHU Lille, 59000 Lille, France; 44 Univ. Lille, CNRS, Inserm, CHU Lille, Institut Pasteur de Lille, U1019-UMR9017-CIIL-Centre d’Infection Et d’Immunité de Lille, 59000 Lille, France; 45 Medical Department, Drammen Hospital, Vestre Viken Hospital Trust, Drammen, Norway; 46 Department of Clinical Science, University of Bergen, Bergen, Norway; 47 Service, de Réanimation Médiale, GHRMSA Hopital Emile Muller, Mulhouse, France; 48 Department of Infectious Diseases, Vestfold Hospital Trust, Tønsberg, Norway; 49 Department of Intensive Care Medicine, Stavanger University Hospital, Stavanger, Norway; 50 Infectious Diseases Unit, Internal Medicine Department, La Paz University Hospital, Madrid, Spain; 51 Centro de Investigación Biomédica en Red de Enfermedades Infecciosas (CIBERINFEC), IdiPAZ, Madrid, Spain; 52 Department of Medicine, Bærum Hospital, Vestre Viken, Bærum, Norway; 53 Department of Anesthesiology and Intensive Care, University Hospital of North Norway, Tromsø, Norway; 54 Service de Médecine Intensive-Réanimation, Hôpital de La Croix - Rousse - HCL, Lyon, France; 55 CREATIS INSERM U1206-CNRS UMR 5220, Lyon, France; 56 Akershus University Hospital, Lørenskog, Norway; 57 Division of Health Services, Department of Global Health, Norwegian Institute of Public Health, Oslo, Norway; 58 ECRIN, Paris, France; 59 Department of Immunology, Oslo University Hospital, Oslo, Norway; 60 Laboratoire de Virologie, Institut Des Agents Infectieux de Lyon, Centre National de Reference Des Virus Respiratoires France Sud, 69317 Hospices Civils de LyonLyon, France; 61 Research Institute of Internal Medicine, Oslo University Hospital, Oslo, Norway; 62 Norwegian Institute of Public Health, Oslo, Norway; 63 Verona University Hospital, Verona, Italy; 64 Université de Paris, IAME, INSERM, 75018 Paris, France; 65 AP-HP, Hôpital Bichat, Service de Maladies Infectieuses Et Tropicales, 75018 Paris, France; 66 Department of Research Support for Clinical Trials, Oslo University Hospital, Oslo, Norway

**Keywords:** COVID-19, Vaccination, Safety, Baricitinib

## Abstract

**Background:**

Baricitinib has shown efficacy in hospitalized patients with COVID-19, but no placebo-controlled trials have focused specifically on severe/critical COVID, including vaccinated participants.

**Methods:**

Bari-SolidAct is a phase-3, multicentre, randomised, double-blind, placebo-controlled trial, enrolling participants from June 3, 2021 to March 7, 2022, stopped prematurely for external evidence. Patients with severe/critical COVID-19 were randomised to Baricitinib 4 mg once daily or placebo, added to standard of care. The primary endpoint was all-cause mortality within 60 days. Participants were remotely followed to day 90 for safety and patient related outcome measures.

**Results:**

Two hundred ninety-nine patients were screened, 284 randomised, and 275 received study drug or placebo and were included in the modified intent-to-treat analyses (139 receiving baricitinib and 136 placebo). Median age was 60 (IQR 49–69) years, 77% were male and 35% had received at least one dose of SARS-CoV2 vaccine. There were 21 deaths at day 60 in each group, 15.1% in the baricitinib group and 15.4% in the placebo group (adjusted absolute difference and 95% CI − 0.1% [− 8·3 to 8·0]). In sensitivity analysis censoring observations after drug discontinuation or rescue therapy (tocilizumab/increased steroid dose), proportions of death were 5.8% versus 8.8% (− 3.2% [− 9.0 to 2.7]), respectively. There were 148 serious adverse events in 46 participants (33.1%) receiving baricitinib and 155 in 51 participants (37.5%) receiving placebo. In subgroup analyses, there was a potential interaction between vaccination status and treatment allocation on 60-day mortality. In a subsequent post hoc analysis there was a significant interaction between vaccination status and treatment allocation on the occurrence of serious adverse events, with more respiratory complications and severe infections in vaccinated participants treated with baricitinib. Vaccinated participants were on average 11 years older, with more comorbidities.

**Conclusion:**

This clinical trial was prematurely stopped for external evidence and therefore underpowered to conclude on a potential survival benefit of baricitinib in severe/critical COVID-19. We observed a possible safety signal in vaccinated participants, who were older with more comorbidities. Although based on a post-hoc analysis, these findings warrant further investigation in other trials and real-world studies.

*Trial registration* Bari-SolidAct is registered at NCT04891133 (registered May 18, 2021) and EUClinicalTrials.eu (2022-500385-99-00).

**Supplementary Information:**

The online version contains supplementary material available at 10.1186/s13054-022-04205-8.

## Background

Baricitinib is an oral Janus kinase (JAK) 1/2 inhibitor, approved by the European Medicines Agency (EMA) for several chronic autoimmune diseases [1]. Early in the pandemic, baricitinib was suggested as COVID-19 therapy through anti-inflammatory effects by inhibiting the JAK-pathway and antiviral properties by inhibiting receptor-mediated endocytosis [2].

Five randomised controlled trials (RCTs) of baricitinib in COVID-19 have been published with promising results, although study design and effect estimates have varied [3, 4]. The ACTT2-trial met the primary endpoint of reduction in time to recovery with baricitinib plus remdesivir compared with remdesivir alone, although only a minority of the participants received glucocorticoids [5]. The ACTT4-trial comparing remdesivir and baricitinib with remdesivir and dexamethasone found no difference in mechanical ventilation-free survival by day 29. However, dexamethasone was associated with more adverse, treatment-related, and severe/life-threatening adverse events [6].

The manufacturer-sponsored double-blind, placebo-controlled COV-BARRIER trial investigated baricitinib or placebo added to standard of care (SoC), with approximately 80% receiving systemic corticosteroids. The trial failed to show a difference in the primary endpoint, occurrence of disease progression to high-flow oxygen/non-invasive ventilation, invasive mechanical ventilation, or death by day 28. However, a significant decrease of 28-day mortality in baricitinib recipients was observed, particularly in severe disease [7]. A subsequent addendum that included 101 critically ill patients on invasive mechanical ventilation or extracorporeal membrane oxygenation (ECMO), showed a marked reduction in 28-day all-cause mortality from 58% in the placebo group to 39% in the baricitinib group [8]. In the open-label RECOVERY trial, a modest, yet significant effect on mortality was reported, with 28-day mortality reduced from 14% in the control group to 12% in the baricitinib group [9]. Finally in the pragmatic PANCOVID-trial, 28-day survival was not significantly different in participants treated with baricitinib added to SoC versus SoC alone, although its small sample size precluded a definitive conclusion [10].

EU-SolidAct is a pan-European multicentre, adaptive platform trial, with its first sub-study Bari-SolidAct investigating baricitinib in patients with severe/critical COVID-19. The primary objective was to evaluate the effect of baricitinib vs. placebo, given in addition to SoC, on the occurrence of death within 60 days.

## Methods

### Study design and participants

EU-SolidAct is an investigator-initiated, randomised adaptive platform trial for COVID-19 and emerging infectious diseases. Data capture is modular depending on epidemic waves and available resources. Bari-SolidAct is a double-blind, randomized placebo-controlled phase 3 trial investigating baricitinib for severe or critical COVID-19. The Master protocol (EU-SolidAct) and subprotocol (Bari-SolidAct) are available on euclinicaltrials.eu.

## Participants

Eligible participants were adults (≥ 18 years), with SARS-CoV2 infection confirmed by a polymerase chain reaction (PCR) no more than 9 days old, admitted to hospital with severe/critical COVID-19, defined as one of the following: (1) SpO2 < 90% on room air, (2) SpO2 90–94% with a downwards trend and/or signs of respiratory distress, (3) need of oxygen by non-invasive ventilation (NIV)/continuous positive airway pressure (CPAP), high-flow oxygen or non-rebreather mask, or iv) need of mechanical ventilation or ECMO.

Key exclusion criteria were suspected serious infection besides COVID-19, recent or recurrent thromboembolism, neutropenia, severe lymphopenia, severe renal dysfunction, pregnancy, breastfeeding, known hypersensitivity to constituents of study drugs, and immunosuppressive drugs including JAK inhibitors, except up to 4 days treatment with corticosteroids for COVID-19.

During the trial, amendments of eligibility criteria included a stricter cut-off for excluding patients with renal dysfunction (from eGFR < 15 to eGFR < 30 mL/min/1.73 m^2^) for consistency with other baricitinib protocols. All participants had eGFR above 30 mL/min/1.73 m^2^ at inclusion. Further amendments in the protocol specific for inclusion of immunocompromized participants with signs of hyperinflammation are reported in the Additional file 1: Online Appendix, as this part of the trial is still open for inclusion. Immunocompromized patients included before the decision to stop inclusion of immunocompetent patients from March 7th 2022 are included in this report, while those enrolled after March 7 are not. This amendment also increased the maximum time from PCR-confirmed SARS-CoV2 test to trial inclusion from 9 to 14 days, and maximum days with COVID-19 symptoms from 14 to 21.

### Ethical considerations

The trial was conducted in accordance with ICH E6 (R2) Good Clinical Practice and the ethical principles of the Declaration of Helsinki. Informed consent by the study participant or legally authorised representative was given prior to inclusion in the study. This is an international trial conducted in several European countries, with approval from ethics committees and national competent authority in each country. The trial has been transferred to CTIS and is now accepted under the Clinical Trial Regulation (CTR), euclinicaltrials.eu (EU CT number 2022-500385-99-00). EU-SolidAct/Bari-SolidAct is also registered at www.clinicaltrials.gov (NCT04891133).

### Randomisation and masking

Eligible patients were randomly allocated to baricitinib or matching placebo in an equal ratio, stratified by study centre and disease severity at baseline. An independent unblinded statistician used a computer-generated randomisation list with a permuted block size of 6 to assign participants to treatment groups and was the only person who knew the block size. All treatment assignments were securely stored on a server with restricted access. Participants, care providers and study personnel were masked to treatment assignment. Baricitinib and matching placebo were provided by Eli Lilly and Company, and drugs were stored, labelled, and shipped to the sites by a pharmaceutical logistics company.

### Procedures

The participants were randomly assigned to one of the following groups: (1) 4 mg baricitinib once daily, or (2) matching placebo. Baricitinib or placebo were administered up to 14 days, and permanently stopped if the patient was discharged from hospital. Baricitinib and placebo were administered orally or by feeding tube. During the study, SoC changed in many countries as a result of updated World Health Organisation (WHO) guidelines recommending tocilizumab for severe and critical COVID-19 [11]. Tocilizumab was prohibited at inclusion in the trial but was allowed as rescue therapy in case of clinical progression.

Participants were assessed for study data, including outcomes and adverse events on days 1 (day of inclusion), 3, 5, 8 and every 7 days if still hospitalised, thereafter until day 90, and at discharge or early discontinuation. Participants were remotely followed up after hospital discharge until day 90. At day 90, participants were asked to answer patient reported outcome measures (PROM) provided via an electronic link. Viral loads and SARS-CoV2 serology were measured centrally from samples of serum and naso/oropharyngeal swabs collected at eligible sites at pre-defined time points during hospitalisation, see Additional file 1: Online Appendix for methods.

### Outcomes

The primary endpoint was occurrence of death within 60 days (measured on day 61 after inclusion). Secondary endpoints were: (1) disease progression on the WHO progression scale within 28 days, (2) time from randomisation to sustained recovery defined as being home and without new complications within 14 days after discharge, (3) time from randomisation to first hospital discharge within 90 days, (4) modified WHO score (mild, moderate, severe or critical disease) on day 14 and 28, (5) occurrence of serious adverse events leading to study treatment discontinuation or death, (6) viral clearance assessed by SARS-CoV-2 PCR in naso/oropharyngeal specimens, (7) markers of systemic inflammation (CRP, ferritin, LDH, D-dimer, procalcitonin) during hospitalisation, and (8) PROM by Oslo COVID-19 QLQ-PW80 sub-scale scores (consisting of 80 items with recall timeframe the last 7 days) at day 90 [12].

### Statistical analysis

Based on mortality rates in the DisCoVeRy [13] and NOR-Solidarity [14] trials, in addition to publicly available statistics from France, we assumed a 60-day mortality probability of 15% in the placebo group, and 10% in the baricitinib group. To show a difference between the treatment groups with a 5% significance level and a 90% power, we calculated that 924 evaluable participants were needed in each group. We planned to randomise 1900 participants in total to account for expected drop-out of 2.7%.

No formal interim analysis for efficacy was planned. Safety assessment by an independent Data Monitoring Committee (DMC) was planned after prespecified number of enrolled participants. Due to external evidence provided by the RECOVERY trial [9], and updated WHO guidelines recommending baricitinib for severe/critical COVID-19 [15], the trial steering committee decided to stop enrolling immunocompetent participants from 7th March 2022, as the present trial was not expected to alter the overall accumulated evidence. This decision was supported by the chair and statistician of the DMC.

Efficacy and safety analyses were performed on the modified intent to treat analysis set, consisting of all randomised participants who received at least one dose of study drug. A sensitivity analysis was performed by censoring post-discontinuation or post-rescue observations for participants who discontinued the study intervention and/or received rescue treatment (tocilizumab or increased dose of systemic corticosteroids).

We analysed the primary endpoint with the Cochran–Mantel–Haenszel test stratified by country/group of neighbouring countries, as the sample size did not allow use of the stratification factors of randomisation (centres and disease severity). For comparison with prior baricitinib trials, a post hoc analysis of death up to day-28 was performed, using the same approach. Kaplan–Meier curves up to day 61 (60 days after inclusion) were plotted.

Additional information of analysis of secondary endpoints including safety are available in the Additional file 1: Online Appendix. The statistical analysis plan (Additional file 1: Online Appendix) was finalised and signed prior to database lock and opening of the randomisation list. All *p* values are 2-sided, with a significance level of 0.05. The analyses were performed using SAS version 9.4 (SAS Institute), and Stata SE version 13 (StataCorp).

### Role of the funding source

The European Commission funded this research, but had no role in design, analysis, interpretation of data, or approval of the manuscript.

## Results

### Participant flow and recruitment

**Fig. 1:**
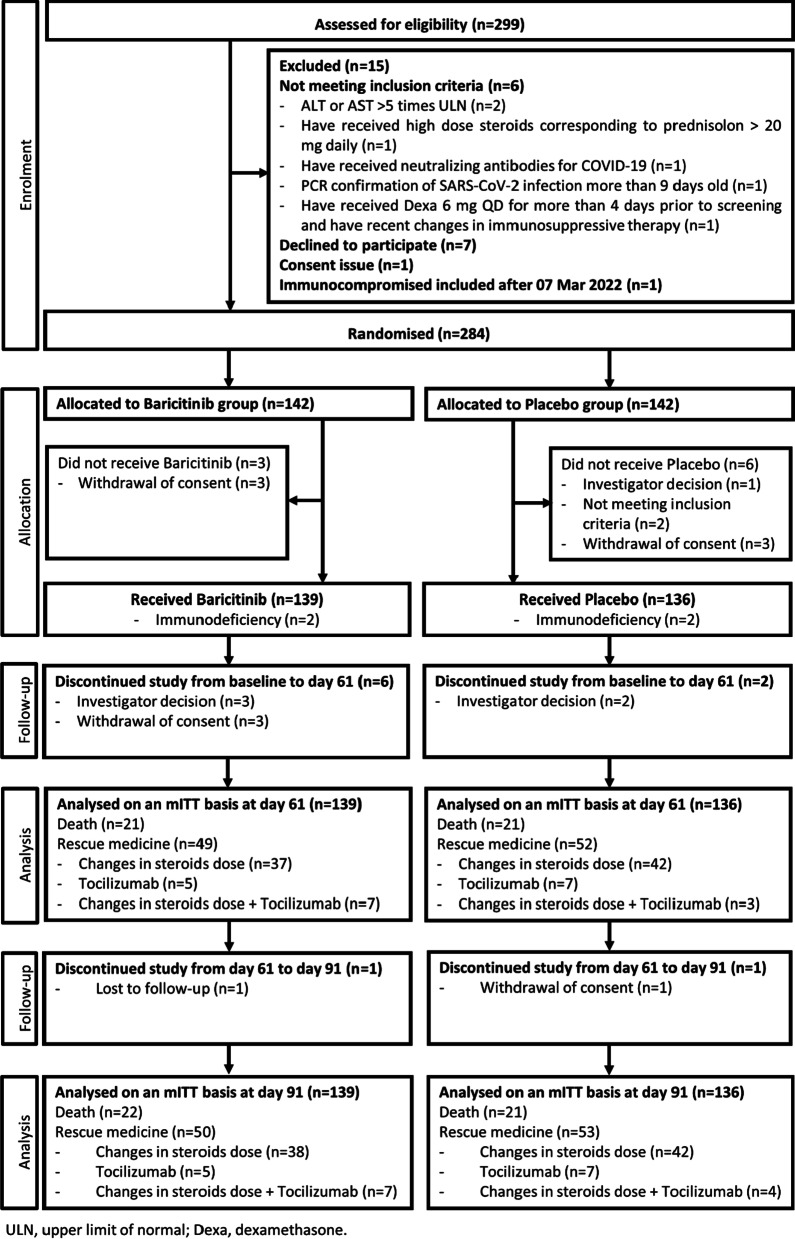
Study Flowchart

The study flowchart is shown in Fig. 1. The participants were recruited between June 3, 2021 and March 7, 2022 at 39 sites (hospital wards and intensive care units, ICUs) in Austria, Belgium, France, Germany, Ireland, Italy, Luxembourg, Norway, Portugal, and Spain (Additional file 1: Table S1). The trial was stopped before reaching the planned sample size due to evidence from the RECOVERY trial indicating survival benefit of baricitinib in the trial population [9]. In total, 299 patients were screened, 284 randomised, 9 participants did not receive any study drug or placebo, and 275 participants were included in both efficacy and safety analyses (139 in the baricitinib group and 136 in placebo).

### Baseline characteristics

Median age was 60 (IQR 49–69) years, 77% were male, 72% had at least one comorbidity, 35% had received at least one dose of SARS-CoV2 vaccine, and 14% had critical disease (mechanical ventilation or ECMO) at inclusion. Systemic corticosteroids were used by 95% of participants, remdesivir by 3% and thromboprophylaxis by 90% (Table 1).

**Table 1:** Baseline demographics and clinical characteristics

	All (*n* = 275)	Baricitinib (*n* = 139)	Placebo (*n* = 136)
*Age (years), median (IQR)*	60 (49–69)	59 (49–68)	60 (50–70)
*n*/*N* (%)			
< 60	137/275 (49.8)	70/139 (50.4)	67/136 (49.3)
≥ 60	138/275 (50.2)	69/139 (49.6)	69/136 (50.7)
*Gender, n/N (%)*			
Male	211/275 (76.7)	112/139 (80.6)	99/136 (72.8)
Female	64/275 (23.3)	27/139 (19.4)	37/136 (27.2)
*Country, n/N (%)*			
Austria	6/275 (2.2)	2/139 (1.4)	4/136 (2.9)
Belgium	8/275 (2.9)	2/139 (1.4)	6/136 (4.4)
France	89/275 (32.4)	48/139 (34.5)	41/136 (30.1)
Ireland	9/275 (3.3)	5/139 (3.6)	4/136 (2.9)
Italy	24/275 (8.7)	12/139 (8.6)	12/136 (8.8)
Luxembourg	1/275 (0.4)	1/139 (0.7)	0/136 (0.0)
Norway	123/275 (44.7)	61/139 (43.9)	62/136 (45.6)
Portugal	3/275 (1.1)	2/139 (1.4)	1/136 (0.7)
Spain	12/275 (4.4)	6/139 (4.3)	6/136 (4.4)
*Comorbidities, n/N (%)*			
Obesity (BMI ≥ 30 kg/m^2^)	99/257 (38.5)	53/127 (41.7)	46/130 (35.4)
Diabetes	61/274 (22.3)	36/139 (25.9)	25/135 (18.5)
Hypertension	85/274 (31.0)	44/139 (31.7)	41/135 (30.4)
Chronic obstructive pulmonary disease	14/274 (5.1)	9/139 (6.5)	5/135 (3.7)
Chronic cardiac disease	52/274 (19.0)	24/139 (17.3)	28/135 (20.7)
Chronic kidney disease	11/274 (4.0)	6/139 (4.3)	5/135 (3.7)
Chronic liver disease	5/274 (1.8)	3/139 (2.2)	2/135 (1.5)
Cancer	10/274 (3.6)	4/139 (2.9)	6/135 (4.4)
Autoimmune disease	12/274 (4.4)	3/139 (2.2)	9/135 (6.7)
Immunodeficiency	4/274 (1.5)	2/139 (1.4)	2/135 (1.5)
*Any comorbidities, n/N (%)*	199/275 (72.4)	107/139 (77.0)	92/136 (67.6)
*Days from first confirmed SARS-CoV-2 PCR to randomisation, median (IQR)*	6 (3–9)	6 (3–8)	7 (3–10)
*Days from symptoms onset to randomisation, median (IQR)*	9 (7–11)	9 (7–11)	10 (8–12)
*n/N (%)*			
≤ 7	74/275 (26.9)	40/139 (28.8)	34/136 (25.0)
8–13	187/275 (68.0)	94/139 (67.6)	93/136 (68.4)
≥ 14	14/275 (5.1)	5/139 (3.6)	9/136 (6.6)
*COVID-19 vaccination, number of injections, n/N (%)*			
0 injection	175/275 (63.6)	87/139 (62.6)	88/136 (64.7)
≥ 1 injection	96/275 (34.9)	49/139 (35.2)	47/136 (34.6)
Unknown	4/275 (1.5)	3/139 (2.2)	1/136 (0.7)
*WHO disease progression score, n/N (%)*			
6 (severe disease)	236/275 (85.8)	121/139 (87.1)	115/136 (84.6)
7–9 (critical disease)	39/275 (14.2)	18/139 (12.9)	21/136 (15.4)
*Concomitant medications, n/N (%)*			
Remdesivir	8/275 (2.9)	6/139 (4.3)	2/136 (1.5)
Systemic corticosteroids	261/275 (94.9)	133/139 (95.7)	128/136 (94.1)
Anticoagulants	250/275 (90.9)	130/139 (93.5)	120/136 (88.2)
*Biochemistry, median (IQR)*			
Lymphocyte count (10^9^ cells/L)			
N	268	135	133
Median (IQR)	0.8 (0.5–1.1)	0.8 (0.6–1.2)	0.7 (0.5–1.0)
Neutrophil count (10^9^ cells/L)			
N	268	135	133
Median (IQR)	6.0 (4.2–8.3)	6.0 (4.2–8.4)	6.0 (4.2–8.3)
C-reactive protein (mg/L)			
N	268	134	134
Median (IQR)	87 (49–140)	77 (35–126)	95 (55–149)
Ferritin (ng/mL)			
N	228	116	112
Median (IQR)	1121 (595–1887)	1039 (609–1887)	1206 (559–1883)
Lactate dehydrogenase (U/L)			
N	235	120	115
Median (IQR)	404 (321–518)	397 (318–526)	436 (323–514)
Procalcitonin (ng/mL)			
N	187	95	92
Median (IQR)	0.2 (0.1–0.3)	0.2 (0.1–0.4)	0.1 (0.1–0.3)
D-dimer (μg/L FEU)			
N	243	119	124
Median (IQR)	900 (600–1500)	844 (620–1360)	904 (594–1592)
*SARS-CoV2 serostatus*			
N	124	65	59
Negative	46/124 (37.1)	21/65 (32.3)	25/59 (42.4)
Positive	78/124 (62.9)	44/65 (67.7)	34/59 (57.6)
Nasopharyngeal viral load, (Log copies/10000 cells) median (IQR)*			
N	124	65	59
Median (IQR)	3.2 (2.1–4.5)	3.2 (2.3–4.5)	3.2 (1.8–4.5)
Viral load value < LOQ	18/124 (14.5)	9/65 (13.9)	9/59 (15.3)

SARS-CoV2 serostatus and nasopharyngeal viral load were done in participants with biobanked samples. SARS-CoV2 serostatus was based on the anti-RBD WT (BAU/mL) value with a cut-off of 10

*Values below the limit of quantification (LOQ) were replaced by the LOQ value (LOQ = 1)

### Efficacy endpoints

**Fig. 2:**
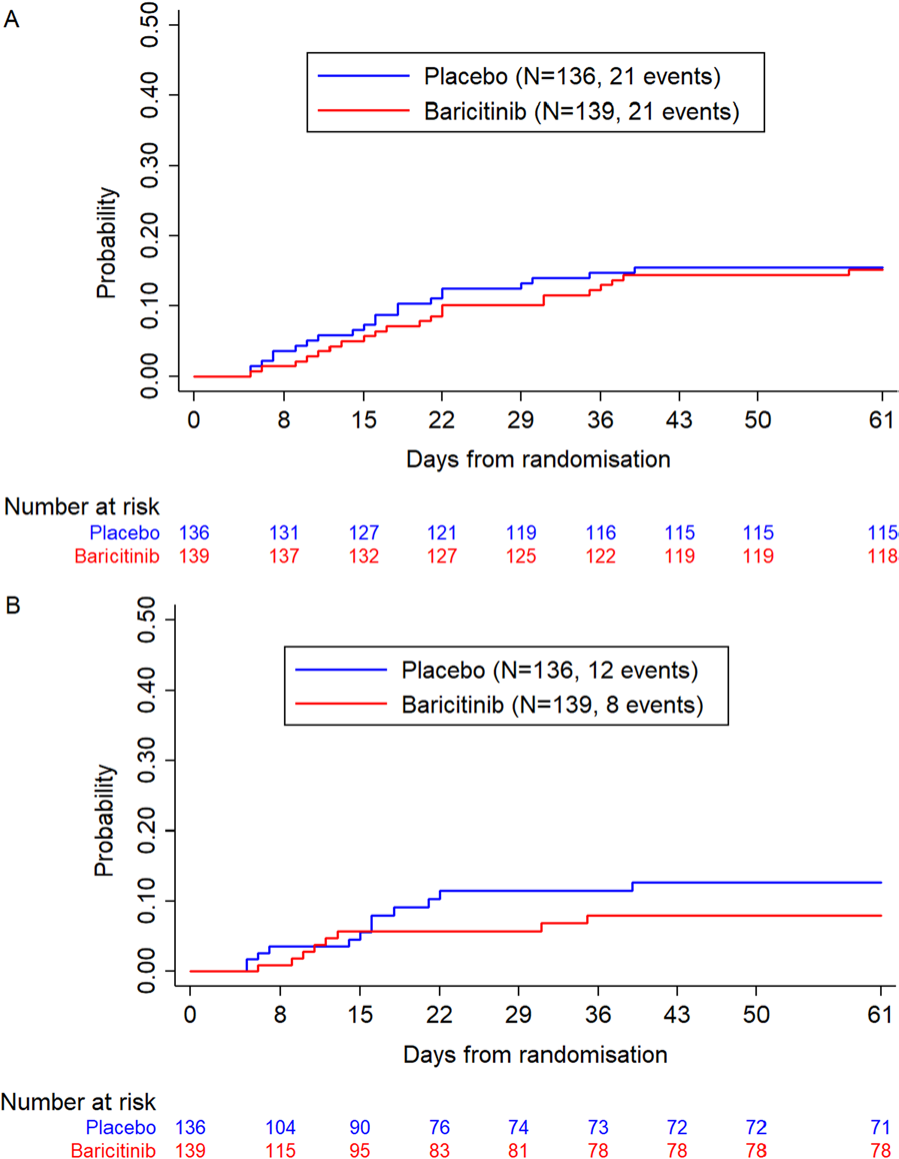
Kaplan–Meier plot of the probability of death within 60 days (measured at day 61 after inclusion), using the modified Intention to Treat population (mITT), consisting of all randomised participants who received at least one dose of study drug; **A** All observations regardless of study intervention discontinuation and/or receipt of rescue therapy; **B** Sensitivity analysis with participants censored at date of rescue therapy or date of discontinuation

Efficacy results are reported in Table 2. There were 21 deaths in each group, leading to a proportion of death at day 60 of 15.1% in the baricitinib group and 15.4% in the placebo group (adjusted absolute difference and 95% confidence intervals (− 0.1% [− 8.3 to 8.0]), while at day 28 the corresponding figures were 10.1% and 13.2% respectively (− 2.9% [− 10.1 to 4.3]). Corresponding Kaplan–Meier plots are shown in Fig. 2A. Figure 2B shows Kaplan–Meier plots of sensitivity analysis when censoring post-discontinuation (6 in the baricitinib group vs 2 in placebo) and post-rescue therapy access observations (49 in the baricitinib group versus 52 in placebo), with proportion of death at day 60 being 5.8% versus 8.8% (− 3.2% [− 9.0 to 2.7]), respectively.

**Table 2:** Primary and secondary efficacy outcomes

	Baricitinib group (*N* = 139)	Placebo group (*N* = 136)	Adjusted Absolute difference (95% CI)	Adjusted OR (95% CI)	P-value*
*Mortality*					
Number of deaths at day 61	21	21			
Proportion (95% CI) at day 61	15.1 (9.6–22.2)	15.4 (9.8–22.6)	− 0.1 (− 8.3–8.0)	0.99 (0.50–1.95)	0.9733
Number of deaths at day 28^+^	14	18			
Proportion (95% CI) at day 28^+^	10.1 (5.6–16.3)	13.2 (8.0–20.1)	− 2.9 (− 10.1–4.3)	0.75 (0.35–1.59)	0.4480
*Disease progression *					
Number of progressions at day 28	25	27			
Proportion (95% CI) at day 28	18.0 (12.0–25.4)	19.9 (13.5–27.6)	− 1.2 (− 10.0–7.4)	0.92 (0.49–1.72)	0.7943

	Baricitinib group (N = 139)	Placebo group (*N* = 136)	Unadjusted sHR or OR (95% CI)	Adjusted sHR or OR (95% CI)	*p* value*
*Sustained recovery*					
Number of recoveries at day 91	107	106			
Cumulative incidence at day 91% (95% CI)	78.8 (72.6–85.4)	79.1 (72.9–85.8)	0.99 (0.76–1.28)	0.98 (0.75–1.28)	0.8604
*Hospital discharge*					
Number of discharges at day 91	111	107			
Cumulative incidence at day 91% (95% CI)	83.8 (77.7–90.3)	79.8 (73.1–87.1)	1.14 (0.88–1.47)	1.14 (0.87–1.48)	0.3490
*Ordinal scale at day 15 – n (%)*			0.94 (0.60–1.48)	0.95 (0.60–1.50)	0.8296
Mild (WHO score 1–3)	81 (58.3)	74 (54.4)			
Moderate (WHO score 4–5)	15 (10.8)	18 (13.2)			
Severe (WHO score 6)	10 (7.2)	8 (5.9)			
Critical (WHO score 7–9)	24 (17.2)	26 (19.1)			
Death (WHO score 10)	25 (6.5)	10 (7.4)			
*Ordinal scale at day 29 – n (%)*			1.13 (0.69–1.84)	1.14 (0.70–1.87)	0.5986
Mild (WHO score 1–3)	96 (69.0)	89 (65.4)			
Moderate (WHO score 4–5)	97 (7.9)	14 (10.3)			
Severe (WHO score 6)	3 (2.2)	4 (3.0)			
Critical (WHO score 7–9)	4 (10.8)	11 (8.1)			
Death (WHO score 10)	14 (10.1)	18 (13.2)			

*p values were obtained using logistic regression or Fine & Gray method with adjustment on stratification factor or proportional odds model

^+^ Post hoc analysis for comparison with other trials

None of the secondary efficacy endpoints (disease progression, sustained recovery, time to first hospital discharge) showed statistically significant differences between the treatment groups (Table 2). As shown in Additional file 1: Table S2, there were no significant differences for any of the PROM subscale scores (Oslo COVID-19 QLQ-PW80), and medians were below 10 for most of the domains (scores ranging from 0 to 100, 100 being the worse). Finally, none of the performed analyses comparing changes in viral loads or systemic inflammation markers were statistically significant, as shown in Additional file 1: Table S3.

### Subgroup analyses

As shown in Additional file 1: Figure S1, no signal of interaction was detected for any of the pre-specified subgroup analyses, except vaccination status (not vaccinated vs at least one dose of SARS-CoV2 vaccine), with a proportion of death at day 60 of 8.0% (7/87) in the baricitinib group versus 15.9% (14/88) in the placebo group in unvaccinated participants, and 26.5% (13/49) versus 14.9% (7/47) respectively in the vaccinated participants (interaction *p* value = 0.0573).

### Safety

The percentages of participants experiencing serious adverse events (SAEs) were similar in both groups: 46 (33.1%) participants with 148 SAEs reported in the baricitinib group, including 41 related to study drug, versus 51 (37.5%) participants with 155 SAEs in the placebo group, including 59 assessed as related to the study drug before unblinding [adjusted incidence rate ratio 0.93 (0.74 to 1.17)] (Table 3). Adverse events of special interest (AESIs) and disease-related events (DREs) were reported in both groups with similar proportions (Additional file 1: Table S4).

**Table 3:** Serious adverse events through day 90

	Baricitinib (*N* = 139)		Placebo (*N* = 136)		Adjusted incidence rate ratio (95% CI)	*p* value*
	Person-months (PM): 349		Person-Months (PM): 349			
	n of events	N of pts (%)	n of events	N of pts (%)		
*Serious adverse events (SAEs)*	148	46 (33.1)	155	51 (37.5)	0.93 (0.74–1.17)	0.550
*Respiratory, thoracic and mediastinal disorders*	51		52			
Acute respiratory distress syndrome	21		21			
Pulmonary embolism	8		13			
Respiratory failure	13		7			
Pneumothorax	3		5			
Hypoxia	1		2			
Respiratory distress	0		2			
Acute respiratory failure	1		0			
Haemoptysis	1		0			
Obstructive airways disorder	1		0			
Pleural effusion	1		0			
Pleuritic pain	1		0			
Pneumonitis	0		1			
Pulmonary hypertension	0		1			
*Infections and infestations*	63		54			
Pneumonia	26		25			
Pneumonia bacterial	10		8			
Septic shock	8		4			
Bacteraemia	4		6			
Bronchopulmonary aspergillosis	0		3			
COVID-19 pneumonia	3		0			
Sepsis	1		2			
Cytomegalovirus infection reactivation	2		0			
Fungaemia	2		0			
Superinfection bacterial	1		1			
Bacterial infection	1		0			
Candida pneumonia	1		0			
Device related bacteraemia	1		0			
Disseminated aspergillosis	0		1			
Hepatitis B reactivation	0		1			
Herpes simplex	1		0			
Infectious pleural effusion	0		1			
Mediastinitis	1		0			
Prostatitis Escherichia coli	0		1			
Septic arthritis staphylococcal	0		1			
Staphylococcal bacteraemia	1		0			
*General disorders and administration site conditions*	8		5			
Multiple organ dysfunction syndrome	6		5			
Chest pain	1		0			
Death	1		0			
*Renal and urinary disorders*	10		13			
Acute renal failure	7		4			
Acute kidney injury	2		6			
Renal failure	1		2			
Chronic kidney disease	0		1			
*Blood and lymphatic system disorders*	0		4			
Anaemia	0		2			
Bicytopenia	0		1			
Thrombocytopenia	0		1			
*Cardiac disorders*	5		5			
Cardiac disorder	2		2			
Cardiac failure	1		1			
Acute coronary syndrome	0		1			
Acute myocardial infarction	0		1			
Bradycardia	1		0			
Myocarditis / pericarditis	1		0			
*Injury, poisoning and procedural complications*	0		1			
Hand fracture	0		1			
*Nervous system disorders*	2		3			
Coma	2		0			
Facial paralysis	0		1			
Haemorrhage intracranial	0		1			
Subarachnoid haemorrhage	0		1			
*Gastrointestinal disorders*	0		3			
Ileus	0		1			
Intestinal ischaemia	0		1			
Rectal haemorrhage	0		1			
*Vascular disorders*	4		4			
Deep vein thrombosis	2		3			
Circulatory collapse	1		0			
Hypotension	1		0			
Peripheral artery thrombosis	0		1			
*Congenital, familial and genetic disorders*	0		1			
Atrial septal defect	0		1			
*Investigations*	5		9			
Transaminases increased	4		8			
Blood bilirubin increased	0		1			
C-reactive protein increased	1		0			
*Musculoskeletal and connective tissue disorders*	0		1			
Spondylolisthesis	0		1			
*Drug-related SAEs*	41	25 (18.0)	59	28 (20.6)	0.77 (0.52–1.16)	0.213
*Respiratory, thoracic and mediastinal disorders*	10		14			
Pulmonary embolism	5		8			
Respiratory failure	2		2			
Acute respiratory distress syndrome	1		2			
Acute respiratory failure	1		0			
Obstructive airways disorder	1		0			
Pneumonitis	0		1			
Pneumothorax	0		1			
*Renal and urinary disorders*	3		6			
Acute kidney injury	2		5			
Chronic kidney disease	0		1			
Renal failure	1		0			
*Infections and infestations*	22		23			
Pneumonia	8		9			
Pneumonia bacterial	5		3			
Bacteraemia	0		3			
Cytomegalovirus infection reactivation	2		0			
Sepsis	1		1			
Superinfection bacterial	1		1			
Bacterial infection	1		0			
Bronchopulmonary aspergillosis	0		1			
COVID-19 pneumonia	1		0			
Candida pneumonia	1		0			
Hepatitis B reactivation	0		1			
Infectious pleural effusion	0		1			
Mediastinitis	1		0			
Prostatitis Escherichia coli	0		1			
Septic arthritis staphylococcal	0		1			
Septic shock	0		1			
Staphylococcal bacteraemia	1		0			
*General disorders and administration site conditions*	1		3			
Multiple organ dysfunction syndrome	1		3			
*Blood and lymphatic system disorders*	0		2			
Anaemia	0		1			
Bicytopenia	0		1			
*Cardiac disorders*	1		1			
Acute coronary syndrome	0		1			
Cardiac failure	1		0			
*Investigations*	2		8			
Transaminases increased	1		7			
Blood bilirubin increased	0		1			
C-reactive protein increased	1		0			
*Vascular disorders*	2		2			
Deep vein thrombosis	1		1			
Circulatory collapse	1		0			
Peripheral artery thrombosis	0		1			

^*^p values were calculated using Poisson regression analysis that accounted for all events and different follow-up duration for each participant

^+^DRE term including 2 PTs, both under the same SOC

Of note, drug-related SAEs in the placebo arm were assessed as such by the investigator before the unblinding of the study

The results of the efficacy subgroup analysis motivated a post hoc analysis to examine the interaction between vaccination status and treatment group on safety, identifying a significant interaction for SAE occurrence: the proportion of participants that experienced SAEs was 25.3% in the baricitinib group versus 37.5% in the placebo group in unvaccinated participants and 46.9% versus 38.3% respectively in the vaccinated participants (interaction *p* value = 0.001). There was no significant interaction with vaccination status regarding occurrence of serious AESIs or serious DREs (Additional file 1: Table S5). The most frequent SAEs driving this difference were increased occurrence of respiratory complications and severe infections in vaccinated participants treated with baricitinib. Vaccinated participants were on average 11 years older and had more comorbidities, in particular diabetes mellitus, hypertension and chronic cardiac conditions (Additional file 1: Table S6).

## Discussion

In this randomised, double-blind, placebo-controlled trial, no statistically significant difference was observed on 60-day mortality in hospitalised patients with severe/critical COVID-19 receiving SoC and either baricitinib or placebo. Of note, the trial was stopped before reaching planned sample size (*n* = 275 analysed versus *n* = 1900 planned) due to external evidence indicating survival benefit of baricitinib in the trial population. The mortality rate estimates at day 28 and day 60 are consistent with prior studies, in particular the day 28 estimate of the RECOVERY trial [5–10].

Whereas Bari-SolidAct included only patients with severe or critical COVID-19, other trials included mixed populations of mild/moderate and severe disease or only critical disease [5–10]. Background SoC, in particular remdesivir and corticosteroids have varied between trials but has not been associated with different treatment effects in subgroup analyses. In contrast to prior double-blind trials, tocilizumab was permitted as rescue therapy in accordance with updated WHO guidelines [15], and investigators were advised to stop baricitinib/placebo for patients receiving rescue therapy to avoid potential triple immunomodulation. This might have had an impact on the primary analysis, and in sensitivity analyses censoring post-rescue or post-discontinuation observations, the point estimates at day 28 and day 60 are closer to the point estimates of other trials (Fig. 2B).

Another notable difference between trial populations is the vaccination coverage. Whereas, due to timing, ACTT-2 and COV-BARRIER trials included no or very few vaccinated participants (no data reported in pubications) [5, 7, 8], the proportion of vaccinated participants was similar in Bari-SolidAct (35%) and RECOVERY (42%) [9]. In subgroup analysis, we found a signal suggesting a potential interaction between vaccination status and treatment allocation on mortality, with results indicating better survival at day 60 in unvaccinated participants treated with baricitinib while a potential opposite effect was seen in vaccinated participants. In the RECOVERY trial, no such interaction was identified [9], although populations are not directly comparable, with only severe/critical COVID-19 included in Bari-SolidAct.

In a subsequent post hoc analysis, there was a significant interaction between vaccination status and occurrence of SAEs, mainly driven by increased occurrence of respiratory complications and severe infections in vaccinated participants treated with baricitinib. In the placebo group, occurrence of SAEs was similar regardless of vaccination status. No safety signals including pulmonary embolism and other adverse events of special interest, were observed in the overall study population. While subgroup analyses must be interpreted with caution, we consider this result a potential safety signal of baricitinib in vaccinated patients. Although we lack a mechanistic explanation, vaccinated patients were on average 11 years older with more cardiometabolic comorbidities, and had lower levels of ferritin and LDH, in line with a recent observational study of vaccinated patients with breakthrough infections requiring hospitalization [16]. We hypothesise that the risk/benefit-ratio of baricitinib might be different in patients with severe/critical COVID-19 depending on SARS-CoV-2 immunisation status, and that underlying host factors such as comorbidities, older age and possibly the capacity to mount an immune response [17] could contribute to such differences.

Compared to other trials reporting 28-day mortality, participants in Bari-SolidAct were followed up to day-90 for efficacy, safety and patient reported outcomes. Pooled data from the TICO-platform reported that 20% of hospitalised patients had clinically significant post discharge-events, underscoring the need for longer follow-up [18]. Finally, Bari-SolidAct includes biobanking, however we observed no between-group differences in changes in viral load or inflammatory markers, despite the hypothesized dual action of baricitinib on viral entry and inflammatory responses [2, 19].

The main limitation of this trial is that due to its limited sample size, efficacy estimates are imprecise with wide confidence intervals. We did not achieve our target sample size because of delay in trial approval in several European countries [20], and because the trial was stopped prematurely as evidence from the RECOVERY trial indicated survival benefit of baricitinib in the trial population [9]. In addition, evolving SoC during the trial, in particular the introduction of tocilizumab, may have had an impact on the primary analysis since many participants had to discontinue intervention early due to rescue therapy. Furthermore, the subgroup analysis by vaccination status has low credibility according to the ICEMAN criteria [21]. Our study also has obvious strengths, in particular granular safety data, with routine registration of concomitant medication, safety lab and detailed follow-up of serious adverse events up to day-90.

## Conclusions

In this prematurely stopped trial, we did not reach a conclusion for the primary endpoint due to lack of statistical power. In sensitivity analyses censoring observations after rescue therapy with tocilizumab or increased dose of corticosteroids, the point estimate is comparable with previous trials. We observed a significant interaction between vaccination status and treatment group on occurrence of SAEs, although this is based on a post hoc analysis and should be interpreted with caution. This potential safety signal should be explored in other trials and real-world studies, before influencing treatment decisions.

## Supplementary Information

**Additional file 1. **Online Appendix.

## Data Availability

Deidentified, individual participant data, along with a data dictionary describing variables in the dataset, will be made available to researchers whose proposed purpose of use is approved by the EU-SolidAct Trial Steering Committee. To request the dataset, please address directly to the corresponding author (marius.troseid@medisin.uio.no) or to Dominique Costagliola (dominique.costagliola@iplesp.upmc.fr) to obtain a data access form. All requests will be evaluated by the Trial Management Team and the EU-SolidAct Trial Steering Committee. For accepted requests, data will be shared after signing a data transfer agreement with the study sponsor. Data will be shared via a secure online procedure. Related documents, such as the study protocol, statistical analysis plan, and informed consent form, will be made available (with publication) on request to the corresponding author. The data will be open access for the informed consent form, protocol, and statistical analysis plan.

## Abbreviations

AE: Adverse event
AESI: Adverse event of special interest
CPAP: Continuous positive airway pressure
DRE: Disease related event
ECMO: Extra corporal membrane oxygenation
eGFR: Estimated glomerular filtration rate
EMA: European Medicines Agencies
NIV: Non-invasive ventilation
PCR: Polymerase chain reaction
PROM: Patient related outcomes measures
SAE: Serious adverse event
SARS-CoV2: Severe acute respiratory coronavirus 2
SoC: Standard of care
WHO: World health organisation

## References

[1] Shi JG, Chen X, Lee F (2014). The pharmacokinetics, pharmacodynamics, and safety of baricitinib, an oral JAK 1/2 inhibitor, in healthy volunteers. J Clin Pharmacol.

[2] Stebbing J, Phelan A, Griffin I (2020). COVID-19: combining antiviral and anti-inflammatory treatments. Lancet Infect Dis.

[3] Kramer A, Prinz C, Fichtner F, et al. Janus kinase inhibitors for the treatment of COVID-19. Cochrane Database Syst Rev. 2022;6:Cd015209. 10.1002/14651858.CD015209 PMC9190191 35695334

[4] Selvaraj V, Finn A, Lal A (2022). Baricitinib in hospitalised patients with COVID-19: A meta-analysis of randomised controlled trials. EClinicalMedicine.

[5] Kalil AC, Patterson TF, Mehta AK, et al. Baricitinib plus Remdesivir for Hospitalized Adults with Covid-19. N Engl J Med. 2020. 10.1056/NEJMoa2031994 PMC7745180 33306283

[6] Wolfe CR, Tomashek KM, Patterson TF, et al. Baricitinib versus dexamethasone for adults hospitalised with COVID-19 (ACTT-4): a randomised, double-blind, double placebo-controlled trial. Lancet Respir Med. 2022. 10.1016/S2213-2600(22)00088-1 PMC9126560 35617986

[7] Marconi VC, Ramanan AV, de Bono S, et al. Efficacy and safety of baricitinib for the treatment of hospitalised adults with COVID-19 (COV-BARRIER): a randomised, double-blind, parallel-group, placebo-controlled phase 3 trial. Lancet Respir Med. 2021. 10.1016/S2213-2600(21)00331-3 PMC8409066 34480861

[8] Ely EW, Ramanan AV, Kartman CE, et al. Efficacy and safety of baricitinib plus standard of care for the treatment of critically ill hospitalised adults with COVID-19 on invasive mechanical ventilation or extracorporeal membrane oxygenation: an exploratory, randomised, placebo-controlled trial. Lancet Respir Med. 2022. 10.1016/S2213-2600(22)00006-6 PMC8813065 35123660

[9] Baricitinib in patients admitted to hospital with COVID-19 (RECOVERY): a randomised, controlled, open-label, platform trial and updated meta-analysis. Lancet. 2022;400:359–68. 10.1016/S0140-6736(22)01109-6 PMC9333998 35908569

[10] Montejano R, de la Calle-Prieto F, Velasco M, et al. Tenofovir Disoproxil Fumarate/Emtricitabine and Baricitinib for Patients at High Risk of Severe COVID-19: The PANCOVID Randomized Clinical Trial. Clin Infect Dis. 2022. 10.1093/cid/ciac628 PMC9384601 35906838

[11] Update to living WHO guideline on drugs for covid-19. Bmj. 2020;371:m4475. 10.1136/bmj.m4475 33214213

[12] Amdal CD, Taylor K, Kuliś D (2022). Health-related quality of life in patients with COVID-19; international development of a patient-reported outcome measure. J Patient Rep Outcomes.

[13] Ader F, Bouscambert-Duchamp M, Hites M (2022). Remdesivir plus standard of care versus standard of care alone for the treatment of patients admitted to hospital with COVID-19 (DisCoVeRy): a phase 3, randomised, controlled, open-label trial. Lancet Infect Dis.

[14] Barratt-Due A, Olsen IC, Nezvalova-Henriksen K (2021). Evaluation of the effects of remdesivir and hydroxychloroquine on viral clearance in COVID-19: a randomized trial. Ann Intern Med.

[15] Update to living WHO guideline on drugs for covid-19. Bmj. 2022;376:o80. 10.1136/bmj.o80 35027394

[16] Lamacchia G, Mazzoni A, Spinicci M, et al. Clinical and immunological features of SARS-CoV-2 breakthrough infections in vaccinated individuals requiring hospitalization. J Clin Immunol. 2022. 10.1007/s10875-022-01325-2 PMC9674730 35809212

[17] Lipsitch M, Krammer F, Regev-Yochay G (2022). SARS-CoV-2 breakthrough infections in vaccinated individuals: measurement, causes and impact. Nat Rev Immunol.

[18] Douin DJ, Siegel L, Grandits G, et al. Evaluating primary endpoints for COVID-19 therapeutic trials to assess recovery. Am J Respir Crit Care Med. 2022. 10.1164/rccm.202112-2836OC PMC9799123 35580040

[19] Richardson P, Griffin I, Tucker C (2020). Baricitinib as potential treatment for 2019-nCoV acute respiratory disease. Lancet.

[20] Diallo A, Trøseid M, Simensen VC (2022). Accelerating clinical trial implementation in the context of the COVID-19 pandemic: challenges, lessons learned and recommendations from DisCoVeRy and the EU-SolidAct EU response group. Clin Microbiol Infect.

[21] Schandelmaier S, Briel M, Varadhan R (2020). Development of the Instrument to assess the Credibility of Effect Modification Analyses (ICEMAN) in randomized controlled trials and meta-analyses. CMAJ.

