# Supplementary material for: Efficacy and safety of baricitinib in hospitalized adults with severe or critical COVID-19 (Bari-SolidAct): a randomised, double-blind, placebo-controlled phase 3 trial
Source: Crit Care. 2023 Jan 10;27:9. doi: 10.1186/s13054-022-04205-8 (PMC9830601; doi:10.1186/s13054-022-04205-8)
Supplement: Supplementary file 1 — Additional file 1. Online Appendix. [file 13054_2022_4205_MOESM1_ESM.docx]

# **Efficacy and safety of baricitinib in hospitalized adults with severe or critical COVID-19 (Bari-SolidAct): a randomised, double-blind, placebo-controlled phase 3 trial**

SUPPLEMENTARY APPENDIX

Contents

EU-SolidAct trial consortium and Study Group 2

Supplementary methods 9

Inclusion and exclusion criteria 12

Safety 15

Supplementary results 17

# Details of the EU-SolidAct Trial Consortium and Study Group

Writing Committee

Marius Trøseid, MD PhD
Section for Clinical Immunology and Infectious Diseases, Oslo University Hospital, Oslo, Norway
Institute of Clinical Medicine, University of Oslo, Oslo, Norway

José R. Arribas, MD PhD

Infectious Diseases Unit, Internal Medicine Department, La Paz University Hospital, IdiPAZ, Madrid, Spain
Centro de Investigación Biomédica en Red de Enfermedades Infecciosas (CIBERINFEC) Madrid, Spain

Lambert Assoumou, PhD
Sorbonne Université, INSERM, Institut Pierre Louis d’Épidémiologie et de Santé Publique (IPLESP), Paris, France

Aleksander Rygh Holten, MD PhD
Department of Acute Medicine, Oslo University Hospital, Oslo, Norway
Institute of Clinical Medicine, University of Oslo, Oslo, Norway

Julien Poissy, MD PhD
Univ. Lille, Inserm U1285, CHU Lille, Pôle de réanimation,  CNRS, UMR 8576 - UGSF - Unité de Glycobiologie Structurale et Fonctionnelle, F-59000 Lille, France

Vida Terzić, PhD
ANRS | Maladies Infectieuses Emergentes, F-75015 Paris, France

Institut national de la santé et de la recherche médicale, INSERM, F-75013 Paris, France

Fulvia Mazzaferri, MD PhD
Division of Infectious Diseases, Department of Diagnostics and Public Health, University of Verona, Verona, Italy

Jesús Rodríguez Baño, MD PhD
Virgen Macarena University Hospital and Department of Medicine, Seville, Spain

University of Sevilla and Biomedicines Institute of Seville (IBiS)/CSIC, Seville, Spain.
CIBERINFEC, Instituto de Salud Carlos III, Madrid, Spain

Joe Eustace, MD PhD
University College Cork, Cork, Ireland

Maya Hites, MD PhD
Brussels University Hospital-Erasme, Brussels, Belgium
Université Libre de Bruxelles, Brussels, Belgium

Michael Joannidis, MD MD
Division of Intensive Care and Emergency Medicine, Department of Internal Medicine, Medical University Innsbruck

José-Artur Paiva, DM PhD
Intensive Care Medicine Department, Centro Hospitalar Universitário Sao Joao, Porto, Portugal

Faculty of Medicine, University of Porto, Porto, Portugal

Jean Reuter, MD PhD
Centre Hospitalier de Luxembourg, Service de Réanimation-Soins Intensifs, L-1210 Luxembourg, Luxembourg

Isabel Püntmann, MD PhD
Institute of Pharmacology, Hospital group Gesundheit Nord gGmbH, Bremen, Germany

Thale D.J.H. Patrick-Brown, MSc
Division of Surgery, Inflammatory Diseases and Transplantation, Oslo University Hospital, Oslo, Norway

Elin Westerheim, MSc
Section for monitoring, Clinical Trial Unit (CTU), Oslo University Hospital, Oslo, Norway

Katerina Nezvalova-Henriksen, PhD
Department of Hematology, Oslo University Hospital and Oslo Hospital Pharmacy, Oslo, Norway

Lydie Beniguel, PhD
Sorbonne Université, INSERM, Institut Pierre Louis d’Épidémiologie et de Santé Publique (IPLESP), Paris, France

Tuva Børresdatter Dahl, PhD
Research Institute of Internal Medicine, Oslo University Hospital, Oslo, Norway
Division of Emergencies and Critical Care, Oslo University Hospital, Oslo, Norway

Maude Bouscambert, PharmD PhD
Laboratoire de Virologie, Institut des Agents Infectieux de Lyon, Centre National de Reference des Virus Respiratoires France Sud, Hospices Civils de Lyon, F-69317, Lyon, France
Université de Lyon, Virpath, CIRI, INSERM U1111, CNRS UMR5308, ENS Lyon, France
Université Claude Bernard Lyon 1, Lyon, France

Monika Halanova, MD PhD
Department of Epidemiology, Faculty of Medicine, Pavol Jozef Šafárik University in Košice, Košice, Slovakia

Zoltán Péterfi, MD PhD

1st Department of Internal Medicine, Division of Infectology, University of Pécs, Pécs, Hungary

Sotirios Tsiodras, MD PhD
National and Kapodistrian University of Athens, Athens, Greece
University Hospital of Athens Attikon, Athens, Greece

Michael Rezek, MD PhD

St. Anne University Hospital, Brno, Czech Republic

Matthias Briel, MD PhD
Swiss Clinical Trial Organisation and Department of Clinical Research, University Hospital Basel and University of Basel, Basel, Switzerland

Serhat Unal, MD PhD
Hacettepe University Hospital, Ankara, Turkey

Martin Schlegel, MD PhD

Department of Anesthesiology and Intensive Care Medicine, Klinikum rechts der Isar, Technische Universität München, München, Germany

Florence Ader, MD PhD
Hospices Civils de Lyon, Département des maladies infectieuses et tropicales, F-69004, Lyon, France
Centre International de Recherche en Infectiologie (CIRI), Inserm 1111, Université Claude Bernard Lyon 1, CNRS, UMR5308, École Normale Supérieure de Lyon, Univ Lyon, F-69007, Lyon, France

Karine Lacombe, MD PhD
Sorbonne Université, Institut Pierre-Louis d'Épidemiologie et de Santé Publique, INSERM, UMR-S1136, IPLESP, Paris, France and Hôpital Saint-Antoine, Service de Maladies Infectieuses et Tropicales, Paris, France

Cecilie Delphin Amdal, PhD
Research support service and Department of Oncology, Oslo University Hospital, Oslo, Norway

Serge Rodrigues, PhD
Sorbonne Université, INSERM, Institut Pierre Louis d’Épidémiologie et de Santé Publique (IPLESP), Paris, France

Kristian Tonby, MD PhD
Deptartment of Infectious diseases, Oslo University Hospital, Oslo, Norway
Institute of Clinical Medicine, University of Oslo, Oslo, Norway

Alexandre Gaudet, MD PhD
Critical Care Center, Department of Intensive Care Medicine, CHU Lille, F-59000 Lille, France
Univ. Lille, CNRS, Inserm, CHU Lille, Institut Pasteur de Lille, U1019-UMR9017-CIIL-Centre d’Infection et d’Immunité de Lille, F-59000 Lille, France

Lars Heggelund, MD PhD

Medical Department, Drammen hospital, Vestre Viken Hospital Trust, Drammen, Norway

Department of Clinical science, University of Bergen, Bergen, Norway

Joy Mootien, MD PhD
Service, de réanimation médiale, GHRMSA Hopital Emile Muller, Mulhouse, France

Asgeir Johannessen, MD PhD

Department of Infectious Diseases, Vestfold Hospital Trust, Tønsberg, Norway

Institute of Clinical Medicine, University of Oslo, Oslo, Norway

Jannicke Horjen Møller, MD
Department of Intensive Care Medicine, Stavanger University Hospital, Stavanger, Norway

Beatriz Diaz Pollan, PhD
Infectious Diseases Unit, Internal Medicine Department, La Paz University Hospital, Madrid, Spain

IdiPAZ, Centro de Investigación Biomédica en Red de Enfermedades Infecciosas (CIBERINFEC), Madrid, Spain

Anders Aune Tveita, MD PhD
Department of medicine, Vestre Viken, Bærum Hospital, Bærum, Norway

Anders Benjamin Kildal, MD PhD

Department of Anesthesiology and Intensive Care, University hospital of North Norway, Tromsø, Norway

Jean-Christophe Richard, MD PhD
Service de médecine intensive-réanimation, Hôpital de la Croix - Rousse - HCL Lyon, France
CREATIS INSERM U1206-CNRS UMR 5220, Lyon, France

Olav Dalgard, MD PhD
Akershus University Hospital, Lørenskog, Norway
Institute of Clinical Medicine, University of Oslo, Oslo, Norway

Victoria Charlotte Simensen, MD

Norwegian Institute of Public Health, Division of Health Services, Department of Global Health, Oslo, Norway

Aliou Baldé, PhD
Sorbonne Université, INSERM, Institut Pierre Louis d’Épidémiologie et de Santé Publique (IPLESP), Paris, France

Lucie de Gastines, PharmD

ANRS | Maladies Infectieuses Emergentes, F-75015 Paris, France

Institut national de la santé et de la recherche médicale, INSERM, F-75013 Paris, France

Marta del Álamo, PhD

ECRIN, Paris, France

Burç Aydin, PhD

ECRIN, Paris, France

Fridtjof Lund-Johansen, PhD
Department of Immunology, Oslo University Hospital, Oslo, Norway

Mary-Anne Trabaud, PhD

Laboratoire de Virologie, Institut des Agents Infectieux de Lyon, Centre National de Reference des Virus Respiratoires France Sud, Hospices Civils de Lyon, F-69317, Lyon, France

Alpha Diallo, MD, MPH
ANRS | Maladies Infectieuses Emergentes, F-75015 Paris, France

Institut national de la santé et de la recherche médicale, INSERM, F-75013 Paris, France

Bente Halvorsen, PhD

Research Institute of Internal Medicine, Oslo University Hospital, Oslo, Norway
Institute of Clinical Medicine, University of Oslo, Oslo, Norway

John-Arne Røttingen, MD PhD
Norwegian Institute of Public Health, Oslo, Norway

Evelina Tacconelli, MD PhD
Verona University Hospital, Verona, Italy
Division of Infectious Diseases, Department of Diagnostics and Public Health, University of Verona, Verona, Italy

Yazdan Yazdanpanah, MD PhD

Université de Paris, IAME, INSERM, F-75018 Paris, France

AP-HP, Hôpital Bichat, Service de maladies infectieuses et tropicales, F-75018 Paris, France

Inge C. Olsen, PhD

Department of Research Support for Clinical Trials, Oslo University Hospital, Oslo, Norway

Dominique Costagliola, PhD
Sorbonne Université, INSERM, Institut Pierre Louis d’Épidémiologie et de Santé Publique (IPLESP), Paris, France

**Data monitoring committee**

Stefano Vella (chair), Guanhua Du, Donata Medaglini, Mike Jacobs, Sylvie van der Werf, Stuart Pocock, Phaik Yeong Cheah, Karen Barnes, Patrick Yeni, Tim Collier

**Trial steering committee**

Dominique Costagliola (chair), Marius Trøseid, José R. Arribas, Evelina Tacconelli, Yazdan Yazdanapanah, Inge C. Olsen, Lambert Assoumou, Aleksander Rygh Holten, Julien Poissy, Vida Terzić, Fulvia Mazzaferri, Jesús Rodríguez Baño, Joe Eustace, Maya Hites, Michael Joannidis, José-Artur Paiva, Jean Reuter, Isabel Püntmann, Thale D.J.H. Patrick-Brown, Elin Westerheim, Katerina Nezvalova-Henriksen, Lydie Beniguel, Maude Bouscambert, Monika Halanova, Zoltán Péterfi, Sotirios Tsiodras, Michael Rezek, Matthias Briel, Serhat Unal, Martin Schlegel, Florence Ader, Gerhard Schneider, Karine Lacombe, Victoria C. Simensen, Bernd Mühlbauer, Aliou Baldé, Lucie de Gastines, Marta del Álamo, Alpha Diallo, John-Arne Røttingen, Tone Ødegård, Gine Bakkehøi

**Drug expert committee**

José R. Arribas (chair), Brigitte Autran (chair), Yazdan Yazdanpanah, John-Arne Røttingen, Inge C. Olsen, Dominique Costagliola, Marius Trøseid, Magnar Bjørås, Maya Hites, Xavier de- Lambellerie, Fulvia Mezzarri, Jeremie Guedj, Helene Esperou, Julia Lumbroso, Tobias Welte, José-Artur Paiva, Victoria C. Simensen

**Independent scientific and ethics board**

Alexandra Calmy, Søren Pischke, Shaun Treweek, Els Goetghebeur, Adelaide Doussau, Laurence Weiss, Frank Hulstaert, Radu Botgros, John-Arne Røttingen, Marta del Alamo, Florence Chung, Julia Lumbroso, Florence Ader, Maya Hites, José R. Arribas, Markus Zeitlinger, Begonya N. Escalera, Chantal Csajka, Clare Williams

**Statistical advisory group**

Alain Amstutz, Aliou Baldé, Corina Silvia Rüegg, Charles Burdet, Clement Massonnaud, Dominique Costagliola,

Drifa Belhadi, France Mentré, Inge Christoffer Olsen, Lambert Assoumou, Massinissa Aroun, Matthias Briel

**Master protocol task force**

France Mentré, Stephan Ehrmann, Helene Espoerou, Charles Burdet

**Data management and statistics team**

Lambert Assoumou, Lydie Beniguel, Aliou Baldé, Serge Rodrigues

**Pharmacovigilance team**

Alpha Diallo, Vida Terzić, Lucie de Gastines

**Monitoring team**

Elin Westerheim, Marta del Álamo, Burç Aydin

**Quality of Life questionnaire team**

Cecilie Delphin Amdal, Ragnhild Sørum Falk, Kristin Bjordal

**EU-SolidAct sponsor team**

Marius Trøseid (chair), Inge C. Olsen (chief of operations), Thale D.J.H. Patrick-Brown (coordinator and drug responsible), Victoria Charlotte Simensen, Gina Bakkehøi, Tone Ødegård

**Laboratory and biobank team**

Maude Bouscambert, Mary Anne Trabaud, Bente Halvorsen, Fridtjof Lund-Johansen, Tuva Børresdatter Dahl

**Study group partner EU-Response**

Andreas Barratt-Due, John-Arne Røttingen

Local investigators and EU-SolidAct study team members

**Norway**

*Oslo University Hospital:* Aleksander Rygh Holten (NCI), Kristian Tonby (deputy NCI), Anne Ma Dyrhol-Riise (PI), Birgitte Stiksrud, Synne Jenum, Magnhild Eide MacPherson, Nikolai Ravn Aarskog, Kjerstin Røstad, Linda Gail Skeie, Åsne Dahl, Jeanette Konstance Steen, Sarah Nur, Filip Segers, Katrine Andersen Korsan, Ashwini Sethupathy, Ann Jorunn Sandstå, Gunn-Janne Paulsen, Thor Ueland, Annika Michelsen, Pål Aukrust. *Akershus University Hospital:* Olav Dalgard (PI), Jan Erik Berdal, Ingunn Melkeraaen, Merete Moen Tollefsen, Jessica Andreassen, Jannicke Dokken. *Vestre Viken Hospital (Drammen):* Lars Heggelund (PI), Karl Erik Müller, Bjørn Martin Woll, Hanne Opsand, Mette Bogen, Linn-Therese Rød, Trude Steinsvik. *Vestfold Hospital Trust Tønsberg:* Asgeir Johannessen (PI), Bjørn Åsheim-Hansen, Randi Haukaas Bjerkreim. *Stavanger University Hospital:* Åse Berg, Jannicke Horjen Møller (PI), Solfrid Moen, Stina Kvalheim, Kristian Strand. *University Hospital of North Norway:* Anders Benjamin Kildal (PI), Berit Gravrok, Vegard Skogen, Elias Myrvoll Lorentzen. *Lovisenberg Hospital:* Simen Walberg Schive (PI), Lasse Rossvoll, Hedda Hoel, Simon Engebråten, Mia Schie Martinsson. *Vestre Viken Hospital (Bærum):* Anders Aune Tveita (PI), Monica Thallinger, Elise Ådnanes. *St. Olavs Hospital Trondheim:* Raisa Hannula (PI), Nina Bremnes, Kristin Liyanarachi, Birgitta Ehrnström, Martin Kvalshaug, Kari Berge, Marte Bygdås, Linda Gustafsson. *Sykehuset Østfold Kalnes:* Saad Aballi (PI)B, Marianne Strand, Britt Andersen. *Norwegian National Steering Commitee:* Pål Aukrust, Andreas Barratt-Due, Katerina Nezvalova Henriksen, Trine Kåsine, Marius Trøseid, Inge Christoffer Olsen, Aleksander Rygh Holten, Kristian Tonby, Anne Ma Dyrhol-Riise, Olav Dalgard, Jan Erik Berdal.

**France**

*University Hospital of Lille:* Julien Poissy (NCI, PI), Raphaël Favory, Saad Nseir, Sebastien Preau, Mercé Jourdain, Alexandre Gaudet, Geoffrey Ledoux, Arthur Durand, Marion Houard, Anne-Sophie Moreau, Anahita Rouzé, Romain Tortuyaux, Guillaume Degouy, Clémentine Levy, Vincent Liu, Nicolas Dognon, Laure Mariller, Claire Delcourte, Zineb Reguig, Amélie Cerf, Marie Cuvelliez, Eric Kipnis, Marielle Boyer-Besseyre, Anne Bignon, Laurie Parmentier, Damia Meddour, Sara Frade. *Bichat Hospital, Paris:* Jean-François Timsit (PI), Yazdan Yazdanpanah (PI), Nathan Peiffer-Smadja (PI), Paul-Henri Wicky, Etienne de Montmollin, Lila Bouadma, Julien Dessajan, Romain Sonneville, Juliette Patrier, Simona Presente, Zmihi Sylia, Christophe Rioux, Michaël Thy, Lio Collias, Yasmine Bouaraba, Nikita Dobremel. *GHMRSA Mulhouse:* Joy Mootien (PI), Anne-Florence Dureau, Pierre Oudeville, Valentin Pointurier, Yannick Rabouel, Laure Stiel, Camille Alzina, Marie Ramstein. *Hôpital Saint-Antoine, Paris*: Hafid Ait-Oufella (PI), Fatima Hamoudi, Tomas Urbina, Karine Lacombe. *Amiens University Hospital:* Yoann Zerbib (PI), Julien Maizel, Celine Wilpotte. *Dijon University Hospital center:* Lionel Piroth (PI), Mathieu Blot, Thibault Sixt, Florian Moretto, Carole Charles, Sandrine Gohier. *Hôpital Louis Mourier, Colombes:* Damien Roux (PI), Camille Le Breton, Coralie Gernez, Ingrid Thiry. *Hôpital de la Croix-Rousse, Lyon:* Jean-Christophe Richard (PI), Loredana Baboi. *Bordeaux University Hospital center:* Denis Malvy (PI), Alexandre Boyer (PI), Pauline Perreau.

**Italy**

*University of Verona:* Evelina Tacconelli (NCI), Fulvia Mazzaferri (Deputy NCI), Maddalena Armellini, Giulia De Luca. Ospedale S. M. *Annunziata Bagno a Ripoli, Firenze:* Massimo Di Pietro (PI), Benedetta Romanin, Michela Brogi. *Brescia Hospital:* Francesco Castelli (PI), Silvia Amadasi. *Pesaro Hospital:* Francesco Barchiesi (PI), Benedetta Canovari. *Luigi Vanvitelli Hospital, Naples:* Nicola Coppola (PI), Mariantonietta Pisaturo, Antonio Russo, Laura Occhiello. *CD Pharma Group:* Francesco Cataldo (CRO).

**Spain**

*La Paz University Hospital, Madrid:* José R. Arribas (Co-chair/PI), Beatriz Diaz Pollan, Marta Mora Rillo

Javier Queiruga, Enrique Seco, Stefan Stewart, Alberto M. Borobia, Paloma Moraga, Rocío Prieto, Irene García, Carlota Rivera, José Luis Narro. *Hospital Universitario Virgen Macarena, Seville:* Jesús Rodríguez-Baño (PI), Natalia Chacón, Sandra de la Rosa, María Macías, Lydia Barrera, Almudena Serna, Virginia Palomo, Maria Isabel García Sánchez, David Gutiérrez, Ana Silva Campos. *Hospital Costa del Sol, Málaga:* Miguel Ángel Gómez Garfia. *Hospital Universitario de Jaén:* Elvira Bonilla Toyos. *Hospital Universitario Virgen de la Victoria:* Judith Sanabria Cabrera, María Isabel Lucena.

**Belgium**

*Hopital Universitaire de Bruxelles/Université Libre de Bruxelles:* Maya Hites (PI), Eva Larranaga Lapique, Pierre Englert, Zineb Khalil, Frédérique Jacobs, Justine Malaise, Odette Mukangenzi, Cinderella Smissaert, Marc Hildebrand, Delphine Martiny, Audrey Vervacke, Axelle Scarnière, Nicolas Yin, Charlotte Michel. *Universitair Ziekenhuis Brussel:* Lucie Seyler (PI), Sabine Allard, Johan Van Laethem, Gil Verschelden, Annelies Meeuwissen, Alex De Waele, Virgini Van Buggenhout, Dora Monteyne, Nils Noppe. *Cliniques Universitaires Saint-Luc:* Leila Belkhir (PI), Jean Cyr Yombi, Julien De Greef, Jean Baptiste Mesland, Léopold De Ghellinck, Valérie Kin, Céline D'Aoust, Anne Bouvier, Anne- Charlotte Dekeister, Estelle Hawia, Adeline Gaillet, Hélène Deshorme, Severine Halleux, Vanessa Galand.

**Portugal**

*Centro Hospitalar Universitário S. João, Porto*: José-Artur Paiva (NCI), Roberto Roncon-Albuquerque (PI). *Centro Hospitalar Universitário de Coimbra*: Luís Linhares Santos (PI). *Centro Hospitalar Universitário Lisboa Central*: César Burgi Vieira (PI). *Centro Clínico Académico, Braga (CTU*): Rosana Magalhaes, Sónia Ferreira, Mariana Bernardo.

**Ireland**

*University College Cork:* Joe Eustace (NCI), Arthur Jackson (PI), Corinna Sadlier, Sarah O'Connell, Matthew Blair, Edmund Manning, Fiona Cusack, Niamh Kelly. *UCC* *Clinical Research Facility:* Hannah Stephenson, Ruben Keane, Aisling Murphy, Michele Cunnane, Fionnuala Keane, Mary-Claire O'Regan. *Beaumont Hospital:* Eoghan de Barra (PI), Aimee McGreal Bellone, Siobhan O'Regan, Patrick Carey, Jeffrey Harte, Peter Coakley, Aoife Heeney, Dorothy Ryan, Gerard Curley, Samuel McConkey, Imran Sulaiman, Richard Costello, Cora McNally, Claire Foley, Sophie Trainor, Benson Jacob, Suchitra Vengathodi. *St. James University Hospital:* Brian Kent (PI), Colm Bergin (PI), Liam Townsend, Colm Kerr, Nalini Panti, Alberto Garcia Sanz, Binny Benny, Edel O Dea, Niamh Galvin, Claire Burke, Aisling Galvin, Sara Aisiyabi, Deepanjali Lobo. *University Hospital Galway:* John Laffey (PI), Bairbre McNicolas, David Cosgrave, J.R Sheehan, Ciprian Nita, Ciara Hanley, Claire Kelly, Maeve Kernan, Jonathan Murray.

**Luxembourg**

*Centre Hospitalier de Luxembourg:* Jean Reuter (NCI), Thérèse Staub (deputy NCI), Thomas Henin, Gaelle Damilot, Tania Bintener, Joelle Colling, Christian Ferretti, Christophe Werer, Pascal Stammet, Pierre Braquet,

Vic Arendt, Esther Calvo, Christian Michaux. *Luxembourg Institute of Health:* Chouaib Mediouni, Ali Znati, Gloria Montanes, Laetitia Garcia.

**Austria**

*Medical University Innsbruck*: Michael Joannidis (NCI), Claudius Thomé (PI), Robert Breitkopf (PI), Andreas Peer, Georg Lehner, Romuald Bellman, Adelheid Ditlbacher, Armin Finkenstedt, Klemens Zotter, Christian Preuss Hernandez, Sasa Rajsic, Barbara Lanthaler. *Universitätsklinik für Innere Medizin II Landeskrankenhaus, Salzburg:* Richard Greil (deputy NCI).

**Hungary***University of Pécs*: Zoltán Péterfi (NCI), Kiss Tamás (deputy NCI), Szilvia Kovácsné-Levang, David Sipos, Agnes Kappéter, Bernadett Halda-Kiss, Edit Madarassi-Papp. *University of Szeged:* Edit Hajdu (PI), Balázs Bende (HECRIN).

**Greece**

*University Hospital of Athens Attikon:* Sotirios Tsiodras (NCI/PI), Thomas Konstantinos, Charalambos Moschopoulos, Eleni Labrou, Maria Tsakona, Ioannis Grigoropoulos. *General Hospital of Athens Evaggelismos:* Anastasia Kotanidou (deputy NCI/PI), Paraskevi Fragkou, Maria Theodorakopoulou, Eugenia Pantazi, Edison Jahai. *Pharmassist Ltd. CRO:* Maria Moukouli, Dimitrios Siafakas.

**Germany**

*Hospital group Gesundheit Nord gGmbH (Bremen):* Bernd Mühlbauer (NCI), Isabel Püntmann (deputy NCI), Rolf Dembinski (PI), Kathrin Stich. *Technische Universität München:* Gerhard Schneider (NPI), Martin Schlegel.

**Czech Republic**

*St. Anne University Hospital:* Michael Rezek (NCI), Andrej Nagy (PI). *CZECRIN, CTU:* Karolína Grodová, Michaela Kubelová, Lenka Součková, Helena Kartáková Švábová, Regina Demlová.

**Slovakia**

*Pavol Jozef Šafárik University in Košice:* Monika Halanova (NCI), Simona Sonderlichová (CTU).

**Turkey**

*Hacettepe University Hospital, Ankara:* Serhat Unal (NCI), A.C. Inkaya (deputy NCI).

**Switzerland**

*University Hospital Basel and University of Basel:* Matthias Briel (NCI).

**Eli Lilly and Company**

*Project consultans:* Stephanie de Bono, Cynthia E. Kartman, David H. Adams. *Statistics:* Brenda Crowe.

# SUPPLEMENTARY METHODS

**Virology methods**

Normalized viral load in samples were measured at randomisation and at days 3, 5, 8, 11, 15±2 and 29±3 after randomisation, in nasopharyngeal swabs collected through validated devices containing flocked swabs and virus transport medium.

To allow the comparison of samples of different qualities (cell richness) we followed the same methodology of normalised viral load developed previously in the context of influenza virus infection^1^. In brief, the normalised SARS-CoV-2 viral load was determined by RT-PCR blinded to treatment group, divided by the number of cells measured (quantification of HPRT-1 housekeeping gene) and expressed in copies per 104 cells. All samples were centralised and analysed in the same laboratory at the National Centre for Viral Respiratory Infections (Hospices Civils de Lyon, France). We estimated the limit of detection to 1 log10 copies/104 cells and all viral loads strictly below limit of detection were considered as censored^2^.

**References.**

1. Duval X, van der Werf S, Blanchon T, et al. Efficacy of oseltamivir-zanamivir combination compared to each monotherapy for seasonal influenza: A randomized placebo-controlled trial. PLoS Med 2010;7(11):e1000362
2. Ader F, Bouscambert-Duchamp M, Hites M, et al. Remdesivir plus standard of care versus standard of care alone for the treatment of patients admitted to hospital with COVID-19 (DisCoVeRy): a phase 3, randomised, controlled, open-label trial. Lancet Infect Dis 2022;22(2):209-21.

**Serology methods**

Antibodies to antigens from SARS-CoV-2 were measured with a multiplexed bead-based assay as described in detail earlier^1^. Briefly, serum diluted 1:1000 was incubated for 1h with bead-based arrays containing full length spike protein (spike-FL) and the receptor-binding domain (RBD). The arrays were labelled with fluorescent anti-human IgG Fc and analysed by flow cytometry. Signal intensity was defined as the median fluorescence intensity (MFI) of beads coupled with viral proteins divided by the MFI of beads with no viral protein (relative MFI, rMFI). A total of 979 pre-pandemic sera and 810 sera from COVID-19 convalescents were analysed to establish cutoffs for sero-positivity. A double cutoff of rMFI of 5 for RBD and Spike-FL was found to yield a specificity of 99.7% and a sensitivity of 95%^2^

**References.**

1. Tran TT, Vaage EB, Mehta A, et al. Titers of antibodies the receptor-binding domain (RBD) of ancestral SARS-CoV-2 are predictive for levels of neutralizing antibodies to multiple variants [Internet]. 2022 [cited 2022 Jul 22];2022.03.26.484261. Available from: <https://www.biorxiv.org/content/10.1101/2022.03.26.484261v2>
2. Jyssum I, Kared H, Tran TT, et al. Humoral and cellular immune responses to two and three doses of SARS-CoV-2 vaccines in rituximab-treated patients with rheumatoid arthritis: a prospective, cohort study. Lancet Rheumatol 2022;4(3):e177–87.

**Statistical Analysis**

The null hypothesis tested in this study was that there is no difference in 60 day mortality between baricitinib and placebo treatment. The alternative hypothesis was that there is a difference. Superiority of baricitinib would be claimed if the null hypothesis is rejected on the 5% significance level and the estimated treatment difference is in favor of baricitinib. There was one formal null hypothesis tested in this trial, and therefore not adjustment for multiplicity. Secondary results must be interpreted as supportive or explorative depending on the nature.

The analyses were stratified by country/group of neighbouring countries as the number of enrolled participants did not allow use of the stratification factors of randomisation (centres and disease severity). The stratification variable was Austria-Belgium-Luxembourg, France, Ireland, Italy, Norway, Spain-Portugal.

The proportion of patients with disease progression, defined as progression from severe (WHO score 6) to critical/death (WHO score 7-10) or from critical (WHO score 7-9) to death within 28 days was analysed using the same approach as for the primary endpoint. Time to sustained recovery and time to first discharge were analysed using competing risk methods with death as competing risk. Cumulative incidence function estimates using the Fine and Gray method, were calculated, adjusted for country/group of neighbouring countries. The ordinal WHO scale at day 14 and 28 were analysed using the proportional odds model adjusted for countries/group of neighbouring countries. We used the last observation carried forward approach to fill in missing observations.

The mean score for each PROM dimension was compared between treatment groups using the nonparametric Man-Whitney test and analysis of variance, adjusted for the countries/group of neighboring countries.

Change from baseline in viral load was analyzed as for inflammatory markers, except considering time as a continuous variable. In addition to stratification factor, the model was also adjusted for the use of remdesivir or monoclonal antibodies. Changes from baseline in inflammatory markers at day 15 and 29 were compared between the 2 treatment groups using mixed models for repeated measures with random effects and spatial power covariance structure, due to unequal time intervals between visits, adjusted for countries/group of neighboring countries. The model included treatment group, time, countries/group of neighboring countries and interaction between treatment group and time, and interaction between countries/group of neighboring countries and time, with time considered as a categorical variable.

Heterogeneity of the treatment effect across subgroups was assessed by including terms for interactions between treatment and subgroup variables in logistic regression models. Interaction tests were conducted to determine whether the effect of treatment varies between subgroups. The subgroup variables included the disease severity (stratification factor), countries/group of neighboring countries and several demographic and baseline specific variables related to the COVID-19 disease (age, gender, immunocompromised status, days from symptom onset to randomisation, at least one dose of COVID-19 vaccine, use of remdesivir, use of systemic corticoids, COVID-19 serostatus (in the subset with biobanked samples), lymphocyte count, hyperinflamation (an elevation of at least two inflammatory markers of (Ferritin>7000 ug/L, LDH>400 U/L, CRP >75 mg/L).

Safety data were summarised by descriptive statistics. The incidence rates of these events were compared between the treatment groups with a Poisson regression analysis that accounted for all events and different follow-up duration for each participant. The same analyses, restricted to drug related events were also performed.

# INCLUSION AND EXCLUSION CRITERIA

During the trial, amendments of eligibility criteria included stricter cut-off for excluding patients with renal dysfunction (from eGFR below 15 to below 30) for consistency with other baricitinib protocols. In addition, the protocol was amended for inclusion of immunocompromised patients, with longer window of SARS CoV2 PCR positivity (from 9 to 14 days) and mandatory elevation of 2 or more inflammatory markers above the following cut-offs in immunocompromized: ferritin > 700 ug/l, lactate dehydrogenase (LDH) > 400 U/L, C-reactive protein (CRP) >75 mg/L. Updated inclusion and exclusion criteria are given below.

**INCLUSION CRITERIA**

All participants must be eligible according to the master protocol inclusion criteria (SolidAct Part B). Only the general inclusion criteria (GI) for severe/critical COVID-19 are applicable:

| 1. > 18 years of age 2. Laboratory-confirmed SARS-CoV-2 infection (new infection or reinfection) as determined by PCR in any specimen not more than 14 days old 3. Admitted to hospital 4. Informed consent by the participant or legally authorized representative.   GI5B: Severe/critical disease state defined as fulfilling at least one of the following criteria:   1. SpO2<90% on room air, or 2. SpO2 90-94% with a downwards trend and/or signs of respiratory distress***,** or 3. Need of oxygen by NIV (CPAP, BIPAP), high flow or non-rebreather mask, or 4. Need of mechanical ventilation/ECMO |
| --- |

*****Persistently increased respiratory rate, use of accessory muscles, inability to complete full sentences. Clinical judgement must be applied to determine whether a low oxygen saturation is indicative of disease progression or severity or is habitual for a given patient (i.e., with underlying chronic lung disease).

NIV=non-invasive ventilation. CPAP= Continuous Positive Airway Pressure, BPAP= Bi-level Positive Airway Pressure, ECMO = extracorporeal membrane oxygenation.

Note: these are based on the same criteria as in the WHO living guidelines recommending corticosteroid treatment for severe and critical COVID-19.

In addition, the following specific inclusion criterion applies to immunocompromised patients:

SI-01. Immunocompromised patients are eligible only if they have elevation of 2 or more inflammatory markers above the following cutoffs:

-Ferritin > 700 ug/L

-LDH > 400 U/L

-CRP >75 mg/dL

**EXCLUSION CRITERIA**

Participants are excluded from the study if any of the following general exclusion criteria (GE) apply:

- GE-01. Anticipated transfer to another non-trial hospital within 72 hours.

In addition, participants are excluded from being eligible for the intervention cohort if any of the additional specific exclusion (SE) criteria below apply:

- SE-01. Patients receiving Janus kinase (JAK) inhibitors (including baricitinib) for any indication at screening.
- SE-20. Have received tocilizumab or sarilumab for any indication 4 weeks prior to screening.

Note: Tocilizumab as rescue therapy will be allowed in patients with clinical progression after inclusion, see section 6.8 concomitant medication. If tocilizumab or other immunosuppressive rescue therapy is started, IMP should be discontinued.

- SE-21. Patients with recent changes in immunosuppressive therapy that could interfere with the potential effect of baricitinib.

Note: An assessment of the total level of immunosuppression, hematological parameters (SE-13 and SE-14), drug half-lives, drug-drug interactions, and underlying medical conditions (SE-22) must be performed as part of the risk/benefit evaluation.

- Recipients of bone marrow transplant or solid organ transplant last 6 months, or with transplant rejection last 6 months, should not be included.
- Organ transplant recipients receiving triple immunosuppression can only be included if the anti-metabolite (mycophenolic acid or mTOR inhibitor) has been temporarily discontinued per clinical practice^10^. IMP should be discontinued once triple immunosuppression is restarted.
- SE-22. Any medical condition that in the opinion of the investigator poses an inacceptable risk of serious infection or aggravation of the medical condition by participating in the trial.

Note: Patients with acute leukemia or history of lymphoma should not be included. Cancer patients under active treatment, HIV positive individuals with detectable HIV-RNA, or other patient group associated with high risk of serious infection or aggravation of the medical condition should only be included if, in the judgement of the investigator, the potential benefit outweighs the potential risk.

- SE-03. Have received dexamethasone 6 mg daily (or alternative regimens with equivalent of corticosteroids) for more than 4 days prior to screening as part of SoC for severe/critical COVID-19
- SE-04. Had COVID-related symptoms > 21 days or hospitalized > 7 days.
- SE-05. Strong inhibitors of organic anion transporter 3 [OAT3] (e.g., probenecid) that cannot be discontinued at study entry.
- SE-07. Have received any live vaccine within 4 weeks before screening, or intend to receive a live vaccine during the study (until day 90 (+/- 14 days)).

Note: Use of non-live (inactivated) vaccinations, including COVID-19 vaccinations, is allowed for all participants.

- SE-08. Are using or will use extracorporeal blood purification (EBP) device to remove proinflammatory cytokines from the blood such as a cytokine absorption or filtering device, for example, CytoSorb®.
- SE-09. Have diagnosis of current active tuberculosis (TB) or, if known, latent TB treated for less than 4 weeks with appropriate anti-tuberculosis therapy per local guidelines (by history only, no screening tests required).
- SE-10. Suspected serious, active bacterial, fungal, viral, or other infection (besides COVID-19) that in the opinion of the investigator could constitute a risk when taking investigational product.
- SE-12. Have a history of venous thromboembolism (VTE) (deep vein thrombosis [DVT] and/or pulmonary embolism [PE]) within 12 weeks prior to randomization or have a history of recurrent (>1) VTE (DVT/PE).
- SE-13. Neutropenia (absolute neutrophil count <1000 cells/microliters).
- SE-14. Lymphopenia (absolute lymphocyte count <200 cells/microliters).
- SE-15. Alanine aminotransferase (ALT) or aspartate aminotransferase (AST) >5 times ULN.
- SE-16. Subjects with estimated glomerular filtration rate (eGFR) (Modification of Diet in Renal Disease [MDRD]) <30 millilitre/minute/1.73 meters squared are excluded.
- SE-17. Known hypersensitivity to baricitinib or any of its excipients.
- SE-18. Are pregnant or breast feeding or intend to become pregnant or breastfeed during the study.

Note: Women of child-bearing potential (WOCBP) can only be included based on a negative pregnancy test and WOCBP must comply with requirements regarding highly effective contraception. Refer to section 10.1 for contraception requirements.

- SE-19 Participation in any therapeutic clinical trials investigating immunomodulators for COVID-19

# SAFETY

**Safety analysis**

The analysis is based on the overall safety data collected from the signing of the informed consent form (ICF) until the follow-up visit: AE all grades, including Adverse Events of Specific Interest (AESIs) and Disease-Related Events (DREs). The events were coded using the medical dictionary for regulatory affairs, version 25.0 and graded according to the Division of AIDS (DAIDS) table for grading the severity of adult and paediatric adverse events, version 2.1, July, 2017.

**Adverse events of special interest (AESI)**

1. Endocarditis / bacteraemia
2. Meningitis / Encephalitis
3. Bacterial pneumonia, including ventilator-associated pneumonia
4. Pulmonary embolism
5. Deep venous thrombosis
6. Arterial thrombosis
7. Liver dysfunction/hepatotoxicity (grade 3 and 4)
8. Reactivation of chronic infection including tuberculosis, herpes simplex, cytomegalovirus, herpes zoster and hepatitis B.
9. Invasive fungal infection, including invasive pulmonary aspergillosis
10. Serious cardiovascular events, including myocardial infarction and stroke.
11. Gastrointestinal bleeding
12. Diverticulitis (including exacerbation of pre-existing diverticular disease)
13. Gastrointestinal perforation

**Disease related events (DRE)**

1. Hyper/Hypoglycaemia
2. Anaemia
3. Acute renal failure
4. Pancreatitis
5. Myocarditis / pericarditis
6. Severe Acute Respiratory Distress Syndrome (ARDS)
7. Pneumothorax
8. Pleural effusion
9. Coma / Confusion

# SUPPLEMENTARY RESULTS

**Figure S1. Subgroup analyses for the mortality end point**

Hyperinflammation is defined as an elevation of at least two inflammatory markers of (Ferritin>700 µg/L, LDH>400 U/L, CRP >75 mg/dL).


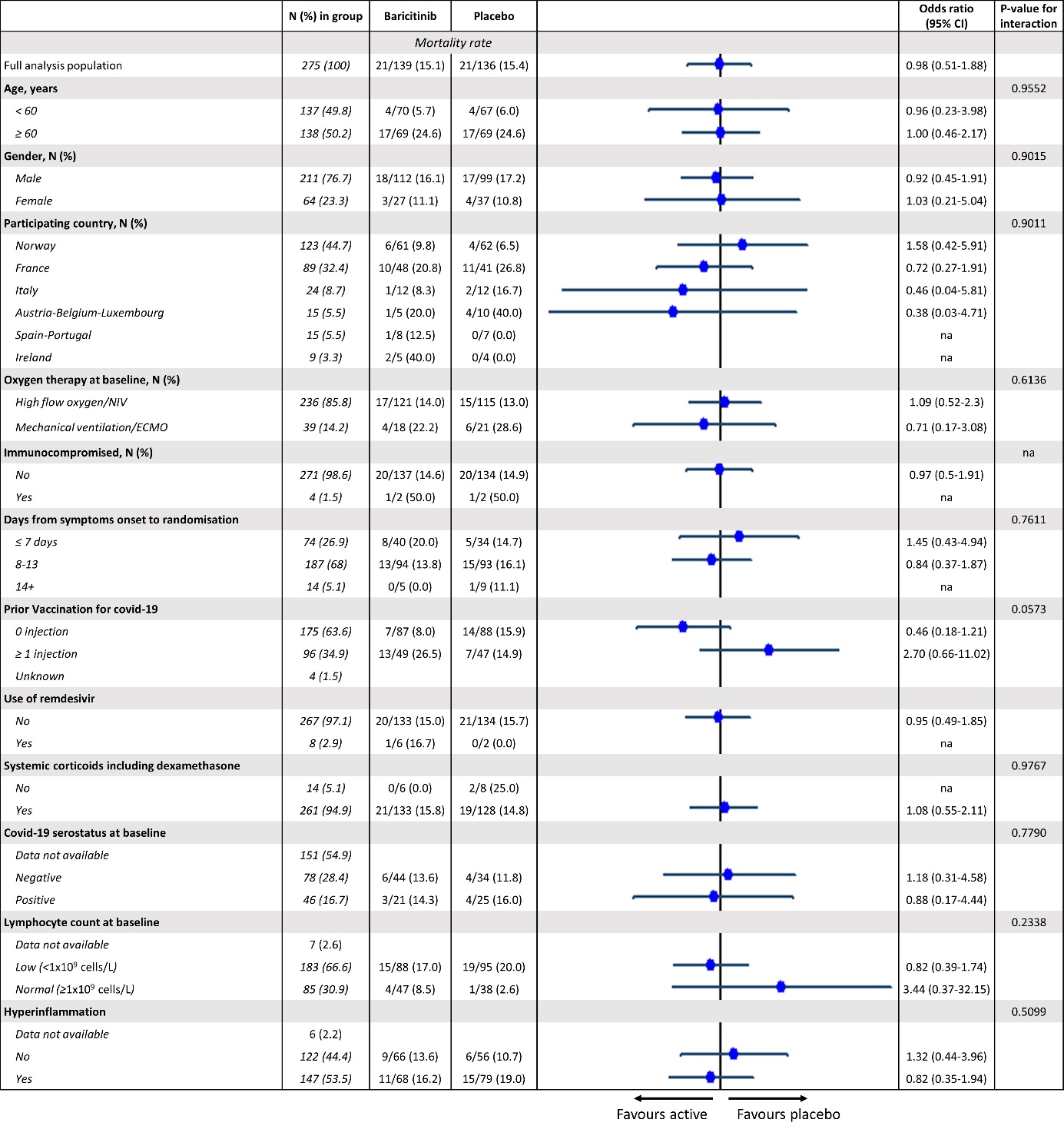


Heterogeneity of the treatment effect across subgroups was assessed by including terms for interactions between treatment and subgroup variables in logistic regression models. Interaction tests were conducted to determine whether the effect of treatment varies between subgroups.

**Table S1. Distribution of enrolled participants according to the participating center**

| **Center number** | **Country** | **Center Name** | **Screened** | **Excluded** | **Never received treatment** | **Analysed** |
| --- | --- | --- | --- | --- | --- | --- |
|  |  |  | N (%) | N (%) | N (%) | N (%) |
| 001 | NORWAY | Akershus Universitetssykehus | 12 (4.0) | 2 (13.3) | 0 (0.0) | 10 (3.6) |
| 002 | NORWAY | Bærum Hospital | 11 (3.7) | 0 (0.0) | 1 (11.1) | 10 (3.6) |
| 003 | NORWAY | Drammen Hospital | 22 (7.4) | 0 (0.0) | 2 (22.3) | 20 (7.3) |
| 005 | NORWAY | Lovisenberg Diaconal Hospital | 5 (1.7) | 0 (0.0) | 0 (0.0) | 5 (1.8) |
| 006 | NORWAY | OUS Ullevål | 25 (8.4) | 0 (0.0) | 0 (0.0) | 25 (9.1) |
| 007 | NORWAY | Stavanger University Hospital | 14 (4.7) | 0 (0.0) | 0 (0.0) | 14 (5.1) |
| 008 | NORWAY | Trondheim University Hospital | 6 (2.0) | 0 (0.0) | 0 (0.0) | 6 (2.2) |
| 009 | NORWAY | University Hospital of North Norway | 10 (3.3) | 0 (0.0) | 0 (0.0) | 10 (3.6) |
| 010 | NORWAY | Vestfold Hospital | 17 (5.7) | 1 (6.7) | 0 (0.0) | 16 (5.8) |
| 011 | NORWAY | Østfold Sykehuset i Kalnes | 7 (2.3) | 0 (0.0) | 0 (0.0) | 7 (2.5) |
| 050 | FRANCE | Hôpital Bichat ID | 4 (1.3) | 0 (0.0) | 0 (0.0) | 4 (1.5) |
| 051 | FRANCE | Hopital Bichat ICU | 6 (2.0) | 0 (0.0) | 0 (0.0) | 6 (2.2) |
| 053 | FRANCE | CHU Amiens Picardie ICU | 9 (3.0) | 1 (6.7) | 0 (0.0) | 8 (2.9) |
| 054 | FRANCE | CHU de Bordeaux / hopital Pellegrin ID | 3 (1.0) | 0 (0.0) | 0 (0.0) | 3 (1.1) |
| 055 | FRANCE | CHU Bordeaux/ Hôpital Pellegrin ICU | 9 (3.0) | 0 (0.0) | 1 (11.1) | 8 (2.9) |
| 056 | FRANCE | CHU François Mitterand | 1 (0.3) | 0 (0.0) | 0 (0.0) | 1 (0.4) |
| 057 | FRANCE | CHU Lille - Hopital Roger Salengro -Pôle Réanimation | 25 (8.4) | 0 (0.0) | 0 (0.0) | 25 (9.1) |
| 058 | FRANCE | GHRMSA Hopital Emile Muller IM | 1 (0.3) | 0 (0.0) | 0 (0.0) | 1 (0.4) |
| 059 | FRANCE | GHRMSA Hopital Emile Muller ICU | 16 (5.4) | 0 (0.0) | 0 (0.0) | 16 (5.8) |
| 061 | FRANCE | Hôpital de la croix-rousse-HCL-ICU | 11 (3.7) | 1 (6.7) | 1 (11.1) | 9 (3.3) |
| 062 | FRANCE | Louis Mourier | 10 (3.3) | 2 (13.3) | 1 (11.1) | 7 (2.5) |
| 064 | FRANCE | Hopital Saint-Antoine | 1 (0.3) | 0 (0.0) | 0 (0.0) | 1 (0.4) |
| 103 | ITALY | ASST - Spedali Civili di Brescia - University of Brescia | 3 (1.0) | 0 (0.0) | 1 (11.1) | 2 (0.7) |
| 105 | ITALY | Azienda Ospedaliera Ospedali Riuniti Marche Nord | 4 (1.3) | 0 (0.0) | 0 (0.0) | 4 (1.5) |
| 114 | ITALY | Ospedale Santa Maria Annunziata | 3 (1.0) | 0 (0.0) | 0 (0.0) | 3 (1.1) |
| 117 | ITALY | Azienda Ospedaliera Universitaria Vanvitelli | 4 (1.3) | 3 (20.0) | 0 (0.0) | 1 (0.4) |
| 118 | ITALY | Azienda Ospedaliera Universitaria Integrata di Verona | 15 (5.0) | 1 (6.7) | 0 (0.0) | 14 (5.1) |
| 154 | GERMANY | Technische Universität München (TUM) | 1 (0.3) | 0 (0.0) | 1 (11.1) | 0 (0.0) |
| 204 | SPAIN | Hospital Universitario La Paz | 13 (4.4) | 0 (0.0) | 1 (11.1) | 12 (4.4) |
| 351 | BELGIUM | Erasme Hospital in Brussels | 4 (1.3) | 0 (0.0) | 0 (0.0) | 4 (1.5) |
| 352 | BELGIUM | Cliniques universitaires Saint-Luc | 4 (1.3) | 0 (0.0) | 0 (0.0) | 4 (1.5) |
| 554 | PORTUGAL | CHLC-HCC - Hospital Curry Cabral, Centro Hospitalar Universitário Lisboa Central | 1 (0.3) | 0 (0.0) | 0 (0.0) | 1 (0.4) |
| 557 | PORTUGAL | CHUC - Centro Hospitalar e Universitário de Coimbra | 2 (0.7) | 0 (0.0) | 0 (0.0) | 2 (0.7) |
| 650 | IRELAND | Beaumont Hospital | 1 (0.3) | 0 (0.0) | 0 (0.0) | 1 (0.4) |
| 651 | IRELAND | Cork University Hospital | 6 (2.0) | 2 (13.3) | 0 (0.0) | 4 (1.5) |
| 655 | IRELAND | Galway University Hospitals | 4 (1.3) | 0 (0.0) | 0 (0.0) | 4 (1.5) |
| 700 | LUXEMBOURG | Centre Hospitalier de Luxembourg | 1 (0.3) | 0 (0.0) | 0 (0.0) | 1 (0.4) |
| 750 | AUSTRIA | Medical University of Innsbruck (EM&CC) | 4 (1.3) | 0 (0.0) | 0 (0.0) | 4 (1.5) |
| 754 | AUSTRIA | Medical University of Innsbruck | 4 (1.3) | 2 (13.3) | 0 (0.0) | 2 (0.7) |
|  |  | **Total** | **299 (100)** | **15 (100)** | **9 (100)** | **275 (100)** |

±

**Table S2:** Patient reported outcome measures (PROM) answered by online link at day 90 (±14), consisting of 80 domains with recall timeframe the last 7 days.

|  | **Baricitinib** | **Placebo** | **P-value*** | **P-value**** |
| --- | --- | --- | --- | --- |
|  | **N=114** | **N=114** |  |  |
| **Temperature** |  |  | 0.2087 | 0.0775 |
| *N* | 85 | 82 |  |  |
| *Median (IQR)* | 0 (0-0) | 0 (0-0) |  |  |
| *[Range]* | [0-50] | [0-83.3] |  |  |
| *Mean (Std)* | 2.8 (8.5) | 5.9 (15.4) |  |  |
| **Fatigue** |  |  | 0.7655 | 0.9513 |
| *N* | 85 | 82 |  |  |
| *Median (IQR)* | 33.3 (11.1-55.6) | 33.3 (0-55.6) |  |  |
| *[Range]* | [0-100] | [0-100] |  |  |
| *Mean (Std)* | 33.7 (27.3) | 33.5 (30.3) |  |  |
| **Malaise** |  |  |  |  |
| *N* | 85 | 82 | 0.5879 | 0.1401 |
| *Median (IQR)* | 8.3 (0-25.0) | 8.3 (0-25.0) |  |  |
| *[Range]* | [0-58.3] | [0-91.7] |  |  |
| *Mean (Std)* | 12.4 (13.3) | 16.6 (20.7) |  |  |
| **Respiratory lower** |  |  |  |  |
| *N* | 85 | 82 | 0.7094 | 0.6113 |
| *Median (IQR)* | 6.7 (0-20.0) | 6.7 (0-20.0) |  |  |
| *[Range]* | [0-73.3] | [0-80.0] |  |  |
| *Mean (Std)* | 14.0 (16.1) | 15.7 (17.6) |  |  |
| **Respiratory upper** |  |  |  |  |
| *N* | 85 | 82 | 0.4832 | 0.8020 |
| *Median (IQR)* | 0 (0-11.1) | 0 (0-11.1) |  |  |
| *[Range]* | [0-66.7] | [0-55.6] |  |  |
| *Mean (Std)* | 6.9 (13.6) | 6.6 (13.2) |  |  |
| **Pain** |  |  |  |  |
| *N* | 85 | 82 | 0.6643 | 0.4095 |
| *Median (IQR)* | 6.7 (0-33.3) | 13.3 (0-33.3) |  |  |
| *[Range]* | [0-73.3] | [0-86.7] |  |  |
| *Mean (Std)* | 17.1 (19.1) | 19.6 (22.5) |  |  |
| **Sensory** |  |  |  |  |
| *N* | 85 | 82 | 0.8441 | 0.6897 |
| *Median (IQR)* | 0 (0-0) | 0 (0-0) |  |  |
| *[Range]* | [0-100] | [0-100] |  |  |
| *Mean (Std)* | 11.0 (23.4) | 9.8 (22.7) |  |  |
| **Neurological** |  |  |  |  |
| *N* | 85 | 82 | 0.1983 | 0.2406 |
| *Median (IQR)* | 0 (0-16.7) | 0 (0-33.3) |  |  |
| *[Range]* | [0-83.3] | [0-100] |  |  |
| *Mean (Std)* | 12.8 (21.6) | 16.7 (23.4) |  |  |
| **Gastrointestinal** |  |  |  |  |
| *N* | 85 | 82 | 0.3256 | 0.1347 |
| *Median (IQR)* | 0 (0-8.3) | 0 (0-8.3) |  |  |
| *[Range]* | [0-41.7] | [0-75] |  |  |
| *Mean (Std)* | 4.4 (8.1) | 6.9 (13.2) |  |  |

The score for the items’ scales were compared between treatment group using the Mann-Whitney test and analysis of variance (ANOVA) with adjustment on the defined stratification factor. The score ranges from 0 to 100, and a higher score represents worse outcome (more symptoms, more problems/worries). *P-values were obtained from a Mann-Whitney test; **Adjusted P-values with ANOVA. Analysis population consists of all modified Intention-to-Treat (mITT) populations for which patient-reported outcome measures were available.

**Table S2-continued:** Patients reported outcomes measures (PROM)

|  | **Baricitinib** | **Placebo** | **P-value*** | **P-value**** |
| --- | --- | --- | --- | --- |
|  | **N=114** | **N=114** |  |  |
| **Emotional functioning** |  |  |  |  |
| *N* | 85 | 82 | 0.5660 | 0.8428 |
| *Median (IQR)* | 8.3 (0-20.8) | 4.2 (0-20.8) |  |  |
| *[Range]* | [0-83.3] | [0-83.3] |  |  |
| *Mean (Std)* | 12.5 (15.2) | 12.2 (16.4) |  |  |
| **Cognitive functioning** |  |  |  |  |
| *N* | 85 | 82 | 0.9148 | 0.9064 |
| *Median (IQR)* | 0 (0-22.2) | 0 (0-22.2) |  |  |
| *[Range]* | [0-100] | [0-77.8] |  |  |
| *Mean (Std)* | 13.7 (20.1) | 12.9 (18.0) |  |  |
| **Physical functioning** |  |  |  |  |
| *N* | 85 | 82 | 0.7262 | 0.8128 |
| *Median (IQR)* | 11.1 (0-22.2) | 11.1 (0-22.2) |  |  |
| *[Range]* | [0-77.8] | [0-100] |  |  |
| *Mean (Std)* | 15.2 (17.7) | 16.1 (22.3) |  |  |
| **Social functioning** |  |  |  |  |
| *N* | 85 | 82 | 0.6235 | 0.9794 |
| *Median (IQR)* | 0 (0-16.7) | 0 (0-16.7) |  |  |
| *[Range]* | [0-66.7] | [0-83.3] |  |  |
| *Mean (Std)* | 12.6 (18.7) | 12.4 (20.9) |  |  |
| **Worries** |  |  |  |  |
| *N* | 85 | 82 | 0.8737 | 0.5942 |
| *Median (IQR)* | 8.3 (4.2-16.7) | 8.3 (0-16.7) |  |  |
| *[Range]* | [0-79.2] | [0-87.5] |  |  |
| *Mean (Std)* | 13.3 (16.4) | 14.1 (17.6) |  |  |
| **Overall quality of life** |  |  |  |  |
| *N* | 85 | 82 | 0.4232 | 0.3769 |
| *Median (IQR)* | 66.7 (50.0-83.3) | 75.0 (58.3-91.7) |  |  |
| *[Range]* | [0-100] | [0-100] |  |  |
| *Mean (Std)* | 68.7 (20.9) | 70.7 (22.1) |  |  |
| **Sleep** |  |  |  |  |
| *N* | 85 | 82 | 0.2195 | 0.2452 |
| *Median (IQR)* | 0 (0-33.3) | 0 (0-33.3) |  |  |
| *[Range]* | [0-100] | [0-100] |  |  |
| *Mean (Std)* | 22.4 (32.7) | 27.6 (33.9) |  |  |
| **Coughed blood** |  |  |  |  |
| *N* | 85 | 82 | 0.9797 | 0.9600 |
| *Median (IQR)* | 0 (0-0) | 0 (0-0) |  |  |
| *[Range]* | [0-33.3] | [0-33.3] |  |  |
| *Mean (Std)* | 0.4 (3.6) | 0.4 (3.7) |  |  |
| **Palpitations** |  |  |  |  |
| *N* | 85 | 82 | **0.0307** | **0.0045** |
| *Median (IQR)* | 0 (0-0) | 0 (0-0) |  |  |
| *[Range]* | [0-33.3] | [0-100] |  |  |
| *Mean (Std)* | 3.5 (10.3) | 11.4 (24.1) |  |  |
| **Blocked nose** |  |  |  |  |
| *N* | 85 | 82 | 0.6173 | 0.6595 |
| *Median (IQR)* | 0 (0-33.3) | 0 (0-33.3) |  |  |
| *[Range]* | [0-100] | [0-100] |  |  |
| *Mean (Std)* | 14.1 (24.3) | 11.8 (21.8) |  |  |
| **Sneezing** |  |  |  |  |
| *N* | 85 | 82 | 0.5928 | 0.5461 |
| *Median (IQR)* | 0 (0-33.3) | 0 (0-33.3) |  |  |
| *[Range]* | [0-100] | [0-100] |  |  |
| *Mean (Std)* | 18.4 (24.4) | 15.9 (21.7) |  |  |

**Table S2-continued:** Patients reported outcomes measures (PROM)

|  | **Baricitinib** | **Placebo** | **P-value*** | **P-value**** |
| --- | --- | --- | --- | --- |
|  | **N=114** | **N=114** |  |  |
| **Red eyes** |  |  |  |  |
| *N* | 85 | 82 | 0.5131 | 0.9842 |
| *Median (IQR)* | 0 (0-0) | 0 (0-0) |  |  |
| *[Range]* | [0-100] | [0-66.7] |  |  |
| *Mean (Std)* | 3.5 (17.1) | 3.3 (12.4) |  |  |
| **Sore eyes** |  |  |  |  |
| *N* | 85 | 82 | 0.1776 | 0.4681 |
| *Median (IQR)* | 0 (0-0) | 0 (0-0) |  |  |
| *[Range]* | [0-100] | [0-100] |  |  |
| *Mean (Std)* | 5.9 (20.0) | 7.7 (18.4) |  |  |
| **Reduced vision** |  |  |  |  |
| *N* | 85 | 82 | 0.4165 | 0.3347 |
| *Median (IQR)* | 0 (0-0) | 0 (0-0) |  |  |
| *[Range]* | [0-100] | [0-100] |  |  |
| *Mean (Std)* | 9.4 (22.2) | 11.4 (23.0) |  |  |
| **Hearing problems** |  |  |  |  |
| *N* | 85 | 82 | 0.9044 | 0.8082 |
| *Median (IQR)* | 0 (0-0) | 0 (0-0) |  |  |
| *[Range]* | [0-66.7] | [0-66.7] |  |  |
| *Mean (Std)* | 4.7 (12.8) | 5.3 (14.3) |  |  |
| **Shaking hands** |  |  |  |  |
| *N* | 85 | 82 | 0.2342 | 0.2852 |
| *Median (IQR)* | 0 (0-0) | 0 (0-33.3) |  |  |
| *[Range]* | [0-100] | [0-100] |  |  |
| *Mean (Std)* | 8.6 (19.4) | 11.8 (21.2) |  |  |
| **Abdominal discomfort** |  |  |  |  |
| *N* | 85 | 82 | 0.1322 | 0.0901 |
| *Median (IQR)* | 0 (0-0) | 0 (0-0) |  |  |
| *[Range]* | [0-66.7] | [0-66.7] |  |  |
| *Mean (Std)* | 3.5 (11.5) | 7.3 (17.4) |  |  |
| **Heartburn** |  |  |  |  |
| *N* | 85 | 82 | 0.7564 | 0.7695 |
| *Median (IQR)* | 0 (0-0) | 0 (0-0) |  |  |
| *[Range]* | [0-66.7] | [0-100] |  |  |
| *Mean (Std)* | 6.7 (16.9) | 7.7 (19.1) |  |  |
| **Vomit** |  |  |  |  |
| *N* | 85 | 82 | 0.9762 | 0.6694 |
| *Median (IQR)* | 0 (0-0) | 0 (0-0) |  |  |
| *[Range]* | [0-66.7] | [0-33.3] |  |  |
| *Mean (Std)* | 1.6 (8.8) | 1.2 (6.3) |  |  |
| **Constipation** |  |  |  |  |
| *N* | 85 | 82 | 0.1897 | 0.4174 |
| *Median (IQR)* | 0 (0-0) | 0 (0-0) |  |  |
| *[Range]* | [0-100] | [0-100] |  |  |
| *Mean (Std)* | 5.9 (20.0) | 6.9 (17.2) |  |  |
| **Dysuria** |  |  |  |  |
| *N* | 85 | 82 | 0.5267 | 0.6082 |
| *Median (IQR)* | 0 (0-0) | 0 (0-0) |  |  |
| *[Range]* | [0-66.7] | [0-66.7] |  |  |
| *Mean (Std)* | 3.1 (12.2) | 4.5 (14.6) |  |  |

**Table S2-continued:** Patients reported outcomes measures (PROM)

|  | **Baricitinib** | **Placebo** | **P-value*** | **P-value**** |
| --- | --- | --- | --- | --- |
|  | **N=114** | **N=114** |  |  |
| **Skin** |  |  |  |  |
| *N* | 85 | 82 | 0.2218 | 0.1299 |
| *Median (IQR)* | 0 (0-33.3) | 0 (0-33.3) |  |  |
| *[Range]* | [0-100] | [0-100] |  |  |
| *Mean (Std)* | 16.1 (27.5) | 21.6 (32.0) |  |  |
| **Hair loss** |  |  |  |  |
| *N* | 85 | 82 | 0.8553 | 0.7509 |
| *Median (IQR)* | 0 (0-33.3) | 0 (0-33.3) |  |  |
| *[Range]* | [0-100] | [0-100] |  |  |
| *Mean (Std)* | 20.8 (36.7) | 19.1 (32.7) |  |  |
| **Coping mentally** |  |  |  |  |
| *N* | 85 | 82 | 0.7588 | 0.9407 |
| *Median (IQR)* | 0 (0-0) | 0 (0-0) |  |  |
| *[Range]* | [0-100] | [0-100] |  |  |
| *Mean (Std)* | 8.6 (20.7) | 7.7 (19.8) |  |  |
| **Confusion** |  |  |  |  |
| *N* | 85 | 82 | 0.0847 | 0.2638 |
| *Median (IQR)* | 0 (0-33.3) | 0 (0-0) |  |  |
| *[Range]* | [0-100] | [0-100] |  |  |
| *Mean (Std)* | 10.6 (19.4) | 6.9 (18.7) |  |  |
| **Heavy housework** |  |  |  |  |
| *N* | 85 | 82 | 0.7760 | 0.8348 |
| *Median (IQR)* | 0 (0-33.3) | 0 (0-33.3) |  |  |
| *[Range]* | [0-100] | [0-100] |  |  |
| *Mean (Std)* | 21.6 (32.8) | 23.6 (34.9) |  |  |
| **Light housework** |  |  |  |  |
| *N* | 85 | 82 | 0.7328 | 0.8907 |
| *Median (IQR)* | 0 (0-0) | 0 (0-0) |  |  |
| *[Range]* | [0-100] | [0-100] |  |  |
| *Mean (Std)* | 9.0 (20.8) | 10.6 (23.4) |  |  |
| **Role functioning** |  |  |  |  |
| *N* | 85 | 82 | 0.6868 | 0.5724 |
| *Median (IQR)* | 0 (0-33.3) | 0 (0-33.3) |  |  |
| *[Range]* | [0-100] | [0-100] |  |  |
| *Mean (Std)* | 25.1 (32.1) | 23.6 (32.5) |  |  |
| **Worry abandoned family/friends** |  |  |  |  |
| *N* | 85 | 82 | 0.1668 | 0.2625 |
| *Median (IQR)* | 0 (0-0) | 0 (0-0) |  |  |
| *[Range]* | [0-66.7] | [0-66.7] |  |  |
| *Mean (Std)* | 0.8 (7.2) | 2.0 (9.6) |  |  |
| **Communication with health care personnel** |  |  |  |  |
| *N* | 85 | 82 | 0.1504 | 0.1339 |
| *Median (IQR)* | 0 (0-0) | 0 (0-0) |  |  |
| *[Range]* | [0-33.3] | [0-100] |  |  |
| *Mean (Std)* | 2.8 (9.2) | 8.1 (21.3) |  |  |

**Table S3:** Viral load and inflammation markers

|  | **Baricitinib (N=139)** | **Placebo (N=136)** | **Difference Mean (SE)** | **P-value** |
| --- | --- | --- | --- | --- |
| **Viral load (log copies/10000 cells), Mean (Standard error [SE])** |  |  |  |  |
| **Day 1** |  |  |  |  |
| N | 65 | 59 |  |  |
| Mean (SE) | 3.3 (0.20) | 3.2 (0.20) | 0.01 (0.27) | 0.978 |
| Day 3 |  |  |  |  |
| N | 60 | 59 |  |  |
| Mean (SE) | 2.7 (0.20) | 2.7 (0.18) |  |  |
| Day 8 |  |  |  |  |
| N | 46 | 36 |  |  |
| Mean (SE) | 2.0 (0.19) | 1.9 (0.24) |  |  |
| Day 15 |  |  |  |  |
| N | 24 | 21 |  |  |
| Mean (SE) | 1.8 (0.26) | 1.4 (0.17) |  |  |
| **Change from baseline in viral load value** |  |  |  |  |
| Mean (Standard error [SE]) change from baseline per additional day | -0.26 (0.09) | -0.27 (0.10) | 0.01 (0.03) | 0.599 |
| **Lymphocytes (10^9 cells/L), Mean (Standard error [SE])** |  |  |  |  |
| Day 1 |  |  |  |  |
| N | 135 | 133 |  |  |
| Mean (SE) | 1.1 (0.15) | 1.0 (0.15) | 0.14 (0.17) | 0.426 |
| Day 8 |  |  |  |  |
| N | 80 | 87 |  |  |
| Mean (SE) | 1.6 (0.17) | 1.6 (0.16) |  |  |
| Day 15 |  |  |  |  |
| N | 47 | 36 |  |  |
| Mean (SE) | 1.6 (0.22) | 1.4 (0.21) |  |  |
| **Change from baseline in lymphocytes value** |  |  |  |  |
| Mean (Standard error [SE]) change from baseline by day 8 | 0.41 (0.18) | 0.59 (0.18) | -0.17 (0.20) | 0.388 |
| Mean (Standard error [SE]) change from baseline by day 15 | 0.70 (0.22) | 0.65 (0.21) | 0.05 (0.25) | 0.842 |
| **Neutrophils (10^9 cells/L), Mean (Standard error [SE])** |  |  |  |  |
| Day 1 |  |  |  |  |
| N | 135 | 133 |  |  |
| Mean (SE) | 6.4 (0.40) | 6.7 (0.39) | -0.36 (0.43) | 0.397 |
| Day 8 |  |  |  |  |
| N | 80 | 87 |  |  |
| Mean (SE) | 9.2 (0.46) | 9.0 (0.44) |  |  |
| Day 15 |  |  |  |  |
| N | 47 | 37 |  |  |
| Mean (SE) | 8.2 (0.62) | 7.9 (0.59) |  |  |
| **Change from baseline in Neutrophils value** |  |  |  |  |
| Mean (Standard error [SE]) change from baseline by day 8 | 2.99 (0.52) | 2.34 (0.51) | 0.65 (0.58) | 0.259 |
| Mean (Standard error [SE]) change from baseline by day 15 | 2.35 (0.70) | 1.59 (0.67) | 0.76 (0.78) | 0.328 |
| **LDH (U/L), Mean (Standard error [SE])** |  |  |  |  |
| Day 1 |  |  |  |  |
| N | 120 | 115 |  |  |
| Mean (SE) | 459 (23.7) | 460 (23.6) | 4.58 (35.37) | 0.897 |
| Day 8 |  |  |  |  |
| N | 71 | 77 |  |  |
| Mean (SE) | 359 (25.9) | 342 (25.1) |  |  |
| Day 15 |  |  |  |  |
| N | 43 | 35 |  |  |
| Mean (SE) | 343 (33.2) | 321 (32.0) |  |  |
| **Change from baseline in LDH value** |  |  |  |  |
| Mean (Standard error [SE]) change from baseline by day 8 | -84.6 (45.0) | -95.1 (43.5) | 10.46 (49.79) | 0.834 |
| Mean (Standard error [SE]) change from baseline by day 15 | -132.6 (53.0) | -127.2 (59.43) | -5.37 (59.43) | 0.928 |

**Table S3 -continued:** Inflammation markers

|  | **Baricitinib (N=139)** | **Placebo (N=136)** | **Difference Mean (SE)** | **P-value** |
| --- | --- | --- | --- | --- |
| **D-Dimer (µg/L FEU), Mean (Standard error [SE])** |  |  |  |  |
| Day 1 |  |  |  |  |
| N | 119 | 124 |  |  |
| Mean (SE) | 1278 (446.3) | 1604 (442.6) | -198.2 (472.6) | 0.675 |
| Day 8 |  |  |  |  |
| N | 71 | 78 |  |  |
| Mean (SE) | 2803 (551.7) | 3562 (490.2) |  |  |
| Day 15 |  |  |  |  |
| N | 41 | 30 |  |  |
| Mean (SE) | 3307 (685.1) | 2578 (659.2) |  |  |
| **Change from baseline in D-Dimer value** |  |  |  |  |
| Mean (Standard error [SE]) change from baseline by day 8 | 1466.0 (614.9) | 1953.6 (595.4) | -487.6 (678.7) | 0.473 |
| Mean (Standard error [SE]) change from baseline by day 15 | 1511.0 (753.2) | 1159.8 (728.7) | 351.2 (843.5) | 0.677 |
| **CRP (mg/L), Mean (Standard error [SE])** |  |  |  |  |
| Day 1 |  |  |  |  |
| N | 134 | 134 |  |  |
| Mean (SE) | 114.0 (19.4) | 123.0 (19.2) | -13.9 (19.5) | 0.478 |
| Day 8 |  |  |  |  |
| N | 80 | 84 |  |  |
| Mean (SE) | 83.4 (22.3) | 42.5 (21.4) |  |  |
| Day 15 |  |  |  |  |
| N | 51 | 41 |  |  |
| Mean (SE) | 138.0 (29.2) | 97.4 (28.1) |  |  |
| **Change from baseline in CRP value** |  |  |  |  |
| Mean (Standard error [SE]) change from baseline by day 8 | -6.2 (25.6) | -45.2 (24.8) | 39.0 (28.3) | 0.170 |
| Mean (Standard error [SE]) change from baseline by day 15 | 57.6 (34.1) | 24.2 (33.0) | 33.4 (38.2) | 0.381 |
| **Procalcitonin (ng/mL), Mean (Standard error [SE])** |  |  |  |  |
| Day 1 |  |  |  |  |
| N | 95 | 92 |  |  |
| Mean (SE) | 0.7 (0.15) | 0.5 (0.13) | 0.12 (0.19) | 0.502 |
| Day 8 |  |  |  |  |
| N | 66 | 68 |  |  |
| Mean (SE) | 0.4 (0.08) | 0.3 (0.09) |  |  |
| Day 15 |  |  |  |  |
| N | 40 | 29 |  |  |
| Mean (SE) | 0.5 (0.10) | 1.0 (0.16) |  |  |
| **Change from baseline in procalcitonin value** |  |  |  |  |
| Mean (Standard error [SE]) change from baseline by day 8 | -0.26 (0.25) | -0.06 (0.24) | -0.22 (0.28) | 0.436 |
| Mean (Standard error [SE]) change from baseline by day 15 | -0.20 (0.29) | 0.29 (0.28) | -0.49 (0.32) | 0.128 |
| **Ferritin (ng/mL), Mean (Standard error [SE])** |  |  |  |  |
| Day 1 |  |  |  |  |
| N | 116 | 112 |  |  |
| Mean (SE) | 1369 (86.1) | 1375 (85.3) | -17.2 (112.3) | 0.879 |
| Day 8 |  |  |  |  |
| N | 66 | 77 |  |  |
| Mean (SE) | 1241 (92.9) | 1067 (82.0) |  |  |
| Day 15 |  |  |  |  |
| N | 41 | 33 |  |  |
| Mean (SE) | 1052 (88.4) | 1088 (140.4) |  |  |
| **Change from baseline in ferritin value** |  |  |  |  |
| Mean (Standard error [SE]) change from baseline by day 8 | -109.2 (99.5) | -140.6 (95.9) | 31.4 (110.0) | 0.776 |
| Mean (Standard error [SE]) change from baseline by day 15 | -226.7 (138.0) | 5.9 (133.2) | -232.6 (154.6) | 0.134 |

**Table S4:** Safety analysis through day 90.

|  | **Baricitinib (N=139)** | | **Placebo (N=136)** | | **Adjusted incidence rate ratio (95% CI)** | **P-value*** |
| --- | --- | --- | --- | --- | --- | --- |
|  | **Person-months (PM): 349** | | **Person-Months (PM): 349** | |  |  |
|  | *n of events* | *N of pts (%)* | *n of events* | *N of pts (%)* |  |  |
| **Serious adverse events (SAEs)** | **148** | **46 (33.1)** | **155** | **51 (37.5)** | **0.93 (0.74-1.17)** | **0.550** |
| ***Respiratory, thoracic and mediastinal disorders*** | **51** |  | **52** |  |  |  |
| *Acute respiratory distress syndrome* | 21 |  | 21 |  |  |  |
| *Pulmonary embolism* | 8 |  | 13 |  |  |  |
| *Respiratory failure* | 13 |  | 7 |  |  |  |
| *Pneumothorax* | 3 |  | 5 |  |  |  |
| *Hypoxia* | 1 |  | 2 |  |  |  |
| *Respiratory distress* | 0 |  | 2 |  |  |  |
| *Acute respiratory failure* | 1 |  | 0 |  |  |  |
| *Haemoptysis* | 1 |  | 0 |  |  |  |
| *Obstructive airways disorder* | 1 |  | 0 |  |  |  |
| *Pleural effusion* | 1 |  | 0 |  |  |  |
| *Pleuritic pain* | 1 |  | 0 |  |  |  |
| *Pneumonitis* | 0 |  | 1 |  |  |  |
| *Pulmonary hypertension* | 0 |  | 1 |  |  |  |
| ***Infections and infestations*** | **63** |  | **54** |  |  |  |
| *Pneumonia* | 26 |  | 25 |  |  |  |
| *Pneumonia bacterial* | 10 |  | 8 |  |  |  |
| *Septic shock* | 8 |  | 4 |  |  |  |
| *Bacteraemia* | 4 |  | 6 |  |  |  |
| *Bronchopulmonary aspergillosis* | 0 |  | 3 |  |  |  |
| *COVID-19 pneumonia* | 3 |  | 0 |  |  |  |
| *Sepsis* | 1 |  | 2 |  |  |  |
| *Cytomegalovirus infection reactivation* | 2 |  | 0 |  |  |  |
| *Fungaemia* | 2 |  | 0 |  |  |  |
| *Superinfection bacterial* | 1 |  | 1 |  |  |  |
| *Bacterial infection* | 1 |  | 0 |  |  |  |
| *Candida pneumonia* | 1 |  | 0 |  |  |  |
| *Device related bacteraemia* | 1 |  | 0 |  |  |  |
| *Disseminated aspergillosis* | 0 |  | 1 |  |  |  |
| *Hepatitis B reactivation* | 0 |  | 1 |  |  |  |
| *Herpes simplex* | 1 |  | 0 |  |  |  |
| *Infectious pleural effusion* | 0 |  | 1 |  |  |  |
| *Mediastinitis* | 1 |  | 0 |  |  |  |
| *Prostatitis Escherichia coli* | 0 |  | 1 |  |  |  |
| *Septic arthritis staphylococcal* | 0 |  | 1 |  |  |  |
| *Staphylococcal bacteraemia* | 1 |  | 0 |  |  |  |
| ***General disorders and administration site conditions*** | **8** |  | **5** |  |  |  |
| *Multiple organ dysfunction syndrome* | 6 |  | 5 |  |  |  |
| *Chest pain* | 1 |  | 0 |  |  |  |
| *Death* | 1 |  | 0 |  |  |  |
| ***Renal and urinary disorders*** | **10** |  | **13** |  |  |  |
| *Acute renal failure* | 7 |  | 4 |  |  |  |
| *Acute kidney injury* | 2 |  | 6 |  |  |  |
| *Renal failure* | 1 |  | 2 |  |  |  |
| *Chronic kidney disease* | 0 |  | 1 |  |  |  |
| ***Blood and lymphatic system disorders*** | **0** |  | **4** |  |  |  |
| *Anaemia* | 0 |  | 2 |  |  |  |
| *Bicytopenia* | 0 |  | 1 |  |  |  |
| *Thrombocytopenia* | 0 |  | 1 |  |  |  |
| ***Cardiac disorders*** | **5** |  | **5** |  |  |  |
| *Cardiac disorder* | 2 |  | 2 |  |  |  |
| *Cardiac failure* | 1 |  | 1 |  |  |  |
| *Acute coronary syndrome* | 0 |  | 1 |  |  |  |
| *Acute myocardial infarction* | 0 |  | 1 |  |  |  |
| *Bradycardia* | 1 |  | 0 |  |  |  |
| *Myocarditis / pericarditis* | 1 |  | 0 |  |  |  |
| ***Injury, poisoning and procedural complications*** | **0** |  | **1** |  |  |  |
| *Hand fracture* | 0 |  | 1 |  |  |  |
| ***Nervous system disorders*** | **2** |  | **3** |  |  |  |
| *Coma* | 2 |  | 0 |  |  |  |
| *Facial paralysis* | 0 |  | 1 |  |  |  |
| *Haemorrhage intracranial* | 0 |  | 1 |  |  |  |
| *Subarachnoid haemorrhage* | 0 |  | 1 |  |  |  |
| ***Gastrointestinal disorders*** | **0** |  | **3** |  |  |  |
| *Ileus* | 0 |  | 1 |  |  |  |
| *Intestinal ischaemia* | 0 |  | 1 |  |  |  |
| *Rectal haemorrhage* | 0 |  | 1 |  |  |  |
| ***Vascular disorders*** | **4** |  | **4** |  |  |  |
| *Deep vein thrombosis* | 2 |  | 3 |  |  |  |
| *Circulatory collapse* | 1 |  | 0 |  |  |  |
| *Hypotension* | 1 |  | 0 |  |  |  |
| *Peripheral artery thrombosis* | 0 |  | 1 |  |  |  |
| ***Congenital, familial and genetic disorders*** | **0** |  | **1** |  |  |  |
| *Atrial septal defect* | 0 |  | 1 |  |  |  |
| ***Investigations*** | **5** |  | **9** |  |  |  |
| *Transaminases increased* | 4 |  | 8 |  |  |  |
| *Blood bilirubin increased* | 0 |  | 1 |  |  |  |
| *C-reactive protein increased* | 1 |  | 0 |  |  |  |
| ***Musculoskeletal and connective tissue disorders*** | **0** |  | **1** |  |  |  |
| *Spondylolisthesis* | 0 |  | 1 |  |  |  |
| **Drug-related SAEs**^+^ | **41** | **25 (18.0)** | **59** | **28 (20.6)** | **0.77 (0.52-1.16)** | **0.213** |
| ***Respiratory, thoracic and mediastinal disorders*** | **10** |  | **14** |  |  |  |
| *Pulmonary embolism* | 5 |  | 8 |  |  |  |
| *Respiratory failure* | 2 |  | 2 |  |  |  |
| *Acute respiratory distress syndrome* | 1 |  | 2 |  |  |  |
| *Acute respiratory failure* | 1 |  | 0 |  |  |  |
| *Obstructive airways disorder* | 1 |  | 0 |  |  |  |
| *Pneumonitis* | 0 |  | 1 |  |  |  |
| *Pneumothorax* | 0 |  | 1 |  |  |  |
| ***Renal and urinary disorders*** | **3** |  | **6** |  |  |  |
| *Acute kidney injury* | 2 |  | 5 |  |  |  |
| *Chronic kidney disease* | 0 |  | 1 |  |  |  |
| *Renal failure* | 1 |  | 0 |  |  |  |
| ***Infections and infestations*** | **22** |  | **23** |  |  |  |
| *Pneumonia* | 8 |  | 9 |  |  |  |
| *Pneumonia bacterial* | 5 |  | 3 |  |  |  |
| *Bacteraemia* | 0 |  | 3 |  |  |  |
| *Cytomegalovirus infection reactivation* | 2 |  | 0 |  |  |  |
| *Sepsis* | 1 |  | 1 |  |  |  |
| *Superinfection bacterial* | 1 |  | 1 |  |  |  |
| *Bacterial infection* | 1 |  | 0 |  |  |  |
| *Bronchopulmonary aspergillosis* | 0 |  | 1 |  |  |  |
| *COVID-19 pneumonia* | 1 |  | 0 |  |  |  |
| *Candida pneumonia* | 1 |  | 0 |  |  |  |
| *Hepatitis B reactivation* | 0 |  | 1 |  |  |  |
| *Infectious pleural effusion* | 0 |  | 1 |  |  |  |
| *Mediastinitis* | 1 |  | 0 |  |  |  |
| *Prostatitis Escherichia coli* | 0 |  | 1 |  |  |  |
| *Septic arthritis staphylococcal* | 0 |  | 1 |  |  |  |
| *Septic shock* | 0 |  | 1 |  |  |  |
| *Staphylococcal bacteraemia* | 1 |  | 0 |  |  |  |
| ***General disorders and administration site conditions*** | **1** |  | **3** |  |  |  |
| *Multiple organ dysfunction syndrome* | 1 |  | 3 |  |  |  |
| ***Blood and lymphatic system disorders*** | **0** |  | **2** |  |  |  |
| *Anaemia* | 0 |  | 1 |  |  |  |
| *Bicytopenia* | 0 |  | 1 |  |  |  |
| ***Cardiac disorders*** | **1** |  | **1** |  |  |  |
| *Acute coronary syndrome* | 0 |  | 1 |  |  |  |
| *Cardiac failure* | 1 |  | 0 |  |  |  |
| ***Investigations*** | **2** |  | **8** |  |  |  |
| *Transaminases increased* | 1 |  | 7 |  |  |  |
| *Blood bilirubin increased* | 0 |  | 1 |  |  |  |
| *C-reactive protein increased* | 1 |  | 0 |  |  |  |
| ***Vascular disorders*** | **2** |  | **2** |  |  |  |
| *Deep vein thrombosis* | 1 |  | 1 |  |  |  |
| *Circulatory collapse* | 1 |  | 0 |  |  |  |
| *Peripheral artery thrombosis* | 0 |  | 1 |  |  |  |
| **Adverse events of special interest (AESI)** | **86** | **44 (31.7)** | **95** | **50 (36.8)** | **0.87 (0.65-1.17)** | **0.346** |
| ***Endocarditis*** | **0** |  | **0** |  |  |  |
| ***Bacteraemia*** | **6** |  | **6** |  |  |  |
| *Bacteraemia* | 4 |  | 6 |  |  |  |
| *Device related bacteraemia* | 1 |  | 0 |  |  |  |
| *Staphylococcal bacteraemia* | 1 |  | 0 |  |  |  |
| ***Meningitis / Encephalitis*** | **0** |  | **0** |  |  |  |
| ***Bacterial pneumonia, including ventilator-associated pneumonia*** | **47** |  | **40** |  |  |  |
| *Bacterial infection* | 2 |  | 0 |  |  |  |
| *Infectious pleural effusion* | 0 |  | 1 |  |  |  |
| *Pneumonia* | 32 |  | 28 |  |  |  |
| *Pneumonia aspiration* | 0 |  | 1 |  |  |  |
| *Pneumonia bacterial* | 12 |  | 9 |  |  |  |
| *Superinfection bacterial* | 1 |  | 1 |  |  |  |
| ***Pulmonary embolism*** | **8** |  | **17** |  |  |  |
| ***Deep venous thrombosis*** | **4** |  | **4** |  |  |  |
| *Catheter site thrombosis* | 0 |  | 1 |  |  |  |
| *Deep vein thrombosis* | 3 |  | 3 |  |  |  |
| *Vena cava thrombosis* | 1 |  | 0 |  |  |  |
| ***Arterial thrombosis*** | **0** |  | **1** |  |  |  |
| *Peripheral artery thrombosis* | 0 |  | 1 |  |  |  |
| ***Hepatotoxicity (grade 3 and 4)*** | **14** |  | **20** |  |  |  |
| *Alanine aminotransferase increased* | 1 |  | 1 |  |  |  |
| *Aspartate aminotransferase increased* | 1 |  | 0 |  |  |  |
| *Blood alkaline phosphatase increased* | 0 |  | 1 |  |  |  |
| *Blood bilirubin increased* | 0 |  | 1 |  |  |  |
| *Cholestasis* | 0 |  | 1 |  |  |  |
| *Gamma-glutamyltransferase increased* | 1 |  | 0 |  |  |  |
| *Hepatocellular injury* | 0 |  | 1 |  |  |  |
| *Hepatotoxicity* | 1 |  | 0 |  |  |  |
| *Transaminases increased* | 10 |  | 15 |  |  |  |
| ***Reactivation of chronic infection*** | **3** |  | **3** |  |  |  |
| *Cytomegalovirus infection reactivation* | 2 |  | 0 |  |  |  |
| *Hepatitis B reactivation* | 0 |  | 1 |  |  |  |
| *Herpes simplex* | 1 |  | 0 |  |  |  |
| *Herpes simplex reactivation* | 0 |  | 2 |  |  |  |
| ***Invasive fungal infection*** | **2** |  | **3** |  |  |  |
| *Bronchopulmonary aspergillosis* | 0 |  | 2 |  |  |  |
| *Disseminated aspergillosis* | 0 |  | 1 |  |  |  |
| *Fungaemia* | 2 |  | 0 |  |  |  |
| ***Serious cardiovascular events*** | **1** |  | **0** |  |  |  |
| *Cardiac failure* | 1 |  | 0 |  |  |  |
| ***Gastrointestinal bleeding*** | **1** |  | **1** |  |  |  |
| *Gastrointestinal haemorrhage* | 1 |  | 0 |  |  |  |
| *Rectal haemorrhage* | 0 |  | 1 |  |  |  |
| ***Diverticulitis*** | **0** |  | **0** |  |  |  |
| ***Gastrointestinal perforation*** | **0** |  | **0** |  |  |  |
| **Drug-related AESI**^+^ | **37** | **29 (20.9)** | **52** | **34 (25.0)** | **0.77 (0.50-1.18)** | **0.225** |
| ***Endocarditis*** | **0** |  | **0** |  |  |  |
| ***Bacteraemia*** | **1** |  | **3** |  |  |  |
| *Bacteraemia* | 0 |  | 3 |  |  |  |
| *Staphylococcal bacteraemia* | 1 |  | 0 |  |  |  |
| ***Meningitis / Encephalitis*** | **0** |  | **0** |  |  |  |
| ***Bacterial pneumonia, including ventilator-associated pneumonia*** | **16** |  | **15** |  |  |  |
| *Bacterial infection* | 2 |  | 0 |  |  |  |
| *Infectious pleural effusion* | 0 |  | 1 |  |  |  |
| *Pneumonia* | 8 |  | 9 |  |  |  |
| *Pneumonia aspiration* | 0 |  | 1 |  |  |  |
| *Pneumonia bacterial* | 5 |  | 3 |  |  |  |
| *Superinfection bacterial* | 1 |  | 1 |  |  |  |
| ***Pulmonary embolism*** | **5** |  | **11** |  |  |  |
| ***Deep venous thrombosis*** | **2** |  | **2** |  |  |  |
| *Catheter site thrombosis* | 0 |  | 1 |  |  |  |
| *Deep vein thrombosis* | 2 |  | 1 |  |  |  |
| ***Arterial thrombosis*** | **0** |  | **1** |  |  |  |
| *Peripheral artery thrombosis* | 0 |  | 1 |  |  |  |
| ***Hepatotoxicity (grade 3 and 4)*** | **10** |  | **16** |  |  |  |
| *Alanine aminotransferase increased* | 1 |  | 1 |  |  |  |
| *Aspartate aminotransferase increased* | 1 |  | 0 |  |  |  |
| *Blood alkaline phosphatase increased* | 0 |  | 1 |  |  |  |
| *Blood bilirubin increased* | 0 |  | 1 |  |  |  |
| *Cholestasis* | 0 |  | 1 |  |  |  |
| *Gamma-glutamyltransferase increased* | 1 |  | 0 |  |  |  |
| *Transaminases increased* | 7 |  | 13 |  |  |  |
| ***Reactivation of chronic infection*** | **2** |  | **3** |  |  |  |
| *Cytomegalovirus infection reactivation* | 2 |  | 0 |  |  |  |
| *Hepatitis B reactivation* | 0 |  | 1 |  |  |  |
| *Herpes simplex reactivation* | 0 |  | 2 |  |  |  |
| ***Invasive fungal infection*** | **0** |  | **1** |  |  |  |
| *Bronchopulmonary aspergillosis* | 0 |  | 1 |  |  |  |
| ***Serious cardiovascular events*** | **1** |  | **0** |  |  |  |
| *Cardiac failure* | 1 |  | 0 |  |  |  |
| ***Gastrointestinal bleeding*** | **0** |  | **0** |  |  |  |
| ***Diverticulitis*** | **0** |  | **0** |  |  |  |
| ***Gastrointestinal perforation*** | **0** |  | **0** |  |  |  |
|  |  |  |  |  |  |  |
| **Any adverse events (AEs) other than SAE and AESI** | **211** | **45 (32.4)** | **204** | **49 (36.0)** | **0.94 (0.77-1.14)** | **0.531** |
|  |  |  |  |  |  |  |
| **Drug-related AEs (any grade) other than SAE and AESI**^+^ | **16** | **11 (7.9)** | **26** | **17 (12.5)** | **0.65 (0.35-1.21)** | **0.175** |
| ***investigations*** | **2** |  | **12** |  |  |  |
| *transaminases increased* | 1 |  | 3 |  |  |  |
| *alanine aminotransferase increased* | 0 |  | 1 |  |  |  |
| *aspartate aminotransferase increased* | 0 |  | 1 |  |  |  |
| *blood creatine phosphokinase increased* | 1 |  | 0 |  |  |  |
| *blood lactic acid increased* | 0 |  | 1 |  |  |  |
| *blood potassium increased* | 0 |  | 1 |  |  |  |
| *blood triglycerides increased* | 0 |  | 1 |  |  |  |
| *gamma-glutamyltransferase increased* | 0 |  | 1 |  |  |  |
| *hepatic enzyme increased* | 0 |  | 1 |  |  |  |
| *liver scan* | 0 |  | 1 |  |  |  |
| *lymphocyte count decreased* | 0 |  | 1 |  |  |  |
| ***infections and infestations*** | **3** |  | **3** |  |  |  |
| *bronchopulmonary aspergillosis* | 0 |  | 2 |  |  |  |
| *urinary tract infection* | 1 |  | 1 |  |  |  |
| *aspergillus infection* | 1 |  | 0 |  |  |  |
| *candida pneumonia* | 1 |  | 0 |  |  |  |
| ***gastrointestinal disorders*** | **2** |  | **1** |  |  |  |
| *diarrhoea* | 1 |  | 1 |  |  |  |
| *vomiting* | 1 |  | 0 |  |  |  |
| ***metabolism and nutrition disorders*** | **0** |  | **3** |  |  |  |
| *hypokalaemia* | 0 |  | 2 |  |  |  |
| *hypoalbuminaemia* | 0 |  | 1 |  |  |  |
| ***psychiatric disorders*** | **0** |  | **3** |  |  |  |
| *restlessness* | 0 |  | 2 |  |  |  |
| *confusional state* | 0 |  | 1 |  |  |  |
| ***renal and urinary disorders*** | **3** |  | **0** |  |  |  |
| *acute kidney injury* | 2 |  | 0 |  |  |  |
| *anuria* | 1 |  | 0 |  |  |  |
| ***musculoskeletal and connective tissue disorders*** | **1** |  | **1** |  |  |  |
| *muscle spasms* | 1 |  | 0 |  |  |  |
| *rhabdomyolysis* | 0 |  | 1 |  |  |  |
| ***respiratory, thoracic and mediastinal disorders*** | **1** |  | **1** |  |  |  |
| *haemoptysis* | 1 |  | 0 |  |  |  |
| *pulmonary congestion* | 0 |  | 1 |  |  |  |
| ***blood and lymphatic system disorders*** | **0** |  | **1** |  |  |  |
| *thrombocytopenia* | 0 |  | 1 |  |  |  |
| ***general disorders and administration site conditions*** | **0** |  | **1** |  |  |  |
| *chest pain* | 0 |  | 1 |  |  |  |
| ***hepatobiliary disorders*** | **1** |  | **0** |  |  |  |
| *cholestasis* | 1 |  | 0 |  |  |  |
| ***injury, poisoning and procedural complications*** | **1** |  | **0** |  |  |  |
| *vasoplegia syndrome* | 1 |  | 0 |  |  |  |
| ***nervous system disorders*** | **1** |  | **0** |  |  |  |
| *dizziness* | 1 |  | 0 |  |  |  |
| ***skin and subcutaneous tissue disorders*** | **1** |  | **0** |  |  |  |
| *alopecia* | 1 |  | 0 |  |  |  |
| **Grade 3-4 AEs other than SAE and AESI** | **31** | **12 (8.6)** | **37** | **16 (11.8)** | **0.73 (0.45-1.19)** | **0.206** |
| ***vascular disorders*** | **11** |  | **4** |  |  |  |
| *hypertension* | 5 |  | 1 |  |  |  |
| *hypotension* | 6 |  | 1 |  |  |  |
| *haemodynamic instability* | 0 |  | 2 |  |  |  |
| ***blood and lymphatic system disorders*** | **4** |  | **7** |  |  |  |
| *lymphopenia* | 3 |  | 3 |  |  |  |
| *thrombocytopenia* | 1 |  | 1 |  |  |  |
| *leukopenia* | 0 |  | 2 |  |  |  |
| *neutropenia* | 0 |  | 1 |  |  |  |
| ***infections and infestations*** | **2** |  | **6** |  |  |  |
| *bronchopulmonary aspergillosis* | 0 |  | 2 |  |  |  |
| *anal abscess* | 0 |  | 1 |  |  |  |
| *aspergillus infection* | 1 |  | 0 |  |  |  |
| *candida pneumonia* | 0 |  | 1 |  |  |  |
| *sepsis* | 1 |  | 0 |  |  |  |
| *septic shock* | 0 |  | 1 |  |  |  |
| *systemic candida* | 0 |  | 1 |  |  |  |
| ***investigations*** | **1** |  | **7** |  |  |  |
| *creatinine renal clearance* | 0 |  | 2 |  |  |  |
| *fibrin d dimer increased* | 0 |  | 2 |  |  |  |
| *lymphocyte count decreased* | 1 |  | 1 |  |  |  |
| *blood albumin decreased* | 0 |  | 1 |  |  |  |
| *blood phosphorus decreased* | 0 |  | 1 |  |  |  |
| ***general disorders and administration site conditions*** | **4** |  | **1** |  |  |  |
| *hyperthermia* | 2 |  | 0 |  |  |  |
| *catheter site haemorrhage* | 1 |  | 0 |  |  |  |
| *oedema* | 0 |  | 1 |  |  |  |
| *pyrexia* | 1 |  | 0 |  |  |  |
| ***metabolism and nutrition disorders*** | **2** |  | **2** |  |  |  |
| *alkalosis* | 0 |  | 1 |  |  |  |
| *hypoalbuminaemia* | 1 |  | 0 |  |  |  |
| *hypocalcaemia* | 0 |  | 1 |  |  |  |
| *metabolic acidosis* | 1 |  | 0 |  |  |  |
| ***gastrointestinal disorders*** | **0** |  | **3** |  |  |  |
| *diarrhoea* | 0 |  | 2 |  |  |  |
| *constipation* | 0 |  | 1 |  |  |  |
| ***respiratory, thoracic and mediastinal disorders*** | **1** |  | **2** |  |  |  |
| *respiratory failure* | 1 |  | 1 |  |  |  |
| *epistaxis* | 0 |  | 1 |  |  |  |
| ***cardiac disorders*** | **0** |  | **2** |  |  |  |
| *atrial fibrillation* | 0 |  | 2 |  |  |  |
| ***hepatobiliary disorders*** | **2** |  | **0** |  |  |  |
| *cholestasis* | 2 |  | 0 |  |  |  |
| ***musculoskeletal and connective tissue disorders*** | **1** |  | **1** |  |  |  |
| *musculoskeletal chest pain* | 0 |  | 1 |  |  |  |
| *rhabdomyolysis* | 1 |  | 0 |  |  |  |
| ***renal and urinary disorders*** | **2** |  | **0** |  |  |  |
| *acute kidney injury* | 1 |  | 0 |  |  |  |
| *anuria* | 1 |  | 0 |  |  |  |
| ***nervous system disorders*** | **0** |  | **1** |  |  |  |
| *neuropathy peripheral* | 0 |  | 1 |  |  |  |
| ***skin and subcutaneous tissue disorders*** | **1** |  | **0** |  |  |  |
| *skin lesion* | 1 |  | 0 |  |  |  |
| ***injury, poisoning and procedural complications*** | **0** |  | **1** |  |  |  |
| *traumatic haemothorax* | 0 |  | 1 |  |  |  |
| **Disease related events (DREs)** | **83** | **83 (59.7)** | **79** | **79 (58.1)** | **0.99 (0.73-1.35)** | **0.963** |
| *ARDS* | 17 |  | 16 |  |  |  |
| *Acute renal failure* | 14 |  | 11 |  |  |  |
| *Anaemia* | 14 |  | 11 |  |  |  |
| *Cardiac disorders* | 5 |  | 7 |  |  |  |
| *Coma / Confusion* | 2 |  | 3 |  |  |  |
| *Hyper/Hypoglycaemia* | 21 |  | 20 |  |  |  |
| *Myocarditis / pericarditis* | 2 |  | 0 |  |  |  |
| *Pancreatitis* | 0 |  | 1 |  |  |  |
| *Pleural effusion* | 4 |  | 1 |  |  |  |
| *Pneumothorax* | 4 |  | 8 |  |  |  |
| *Stroke / Cerebrovascular accident* | 0 |  | 1 |  |  |  |
| **Permanent discontinuation of study treatment** | **24** | **24 (17.3)** | **29** | **29 (21.3)** | **0.83 (0.46-1.47)** | **0.497** |
|  |  |  |  |  |  |  |
| **Death** | **22** | **22 (15.8)** | **21** | **21 (15.4)** | **1.05 (0.55-2.00)** | **0.880** |

*P values were calculated using Poisson regression analysis that accounted for all events and different follow-up duration for each participant.

^+^Of note, drug-related events in the placebo arm were assessed as such by the investigator before the unblinding of the study.

**Table S5:** Safety: subgroup analysis according to vaccination status

|  | **Baricitinib (N=139)** | | **Placebo (N=136)** | | **Incidence rate ratio (95% CI)** | **P-value*** | **P-value for interaction** |
| --- | --- | --- | --- | --- | --- | --- | --- |
|  | **Person-months (PM): 349** | | **Person-Months (PM): 349** | |  |  |  |
|  | *n of events* | *N of pts (%)* | *n of events* | *N of pts (%)* |  |  |  |
| **Serious adverse events (SAEs)** | 148 | 46/139 (33.1) | 155 | 51/136 (37.5) | 0.93 (0.74-1.17) | 0.550 |  |
| ***Vaccination status*** |  |  |  |  |  |  | 0.001 |
| *0 injection* | 73 | 22/87 (25.3) | 103 | 33/88 (37.5) | 0.68 (0.51-0.92) | 0.012 |  |
| ≥*1 injection* | 70 | 23/49 (46.9) | 52 | 18/47 (38.3) | 1.50 (1.05-2.15) | 0.027 |  |
| *Unknown* | 5 | 1/3 | 0 | 0/1 |  |  |  |
| **Drug-related SAEs** | 41 | 25/139 (18.0) | 59 | 28/136 (20.6) | 0.77 (0.52-1.16) | 0.213 |  |
| ***Vaccination status*** |  |  |  |  |  |  | 0.333 |
| *0 injection* | 20 | 14/87 (16.1) | 34 | 17/88 (19.3) | 0.57 (0.33-0.98) | 0.044 |  |
| ≥*1 injection* | 19 | 10/49 (20.4) | 25 | 11/47 (23.4) | 0.85 (0.47-1.54) | 0.584 |  |
| *Unknown* | 2 | 1/3 | 0 | 0/1 |  |  |  |
| **Adverse events of special interest (AESI)** | 86 | 44 (31.7) | 95 | 50 (36.8) | 0.87 (0.65-1.17) | 0.346 |  |
| ***Vaccination status*** |  |  |  |  |  |  | 0.315 |
| *0 injection* | 54 | 29/87 (33.3) | 64 | 34/88 (38.6) | 0.81 (0.57-1.17) | 0.260 |  |
| ≥*1 injection* | 31 | 14/49 (28.6) | 31 | 16/47 (34.0) | 1.11 (0.68-1.83) | 0.672 |  |
| *Unknown* | 1 |  | 0 |  |  |  |  |
| **Drug-related AESI** | 37 | 29 (20.9) | 52 | 34 (25.0) | 0.77 (0.50-1.18) | 0.225 |  |
| ***Vaccination status*** |  |  |  |  |  |  | 0.514 |
| *0 injection* | 23 | 20/87 (23.0) | 35 | 22/88 (25.0) | 0.63 (0.37-1.07) | 0.088 |  |
| ≥*1 injection* | 13 | 8/49 (16.3) | 17 | 12/47 (25.5) | 0.85 (0.41-1.75) | 0.663 |  |
| *Unknown* | 1 |  | 0 |  |  |  |  |
| **Any adverse events (AEs) other than SAE and AESI** | 211 | 45 (32.4) | 204 | 49 (36.0) | 0.94 (0.77-1.14) | 0.531 |  |
| ***Vaccination status*** |  |  |  |  |  |  | 0.155 |
| *0 injection* | 142 | 26/87 (29.9) | 120 | 28/88 (31.2) | 1.14 (0.89-1.45) | 0.294 |  |
| ≥*1 injection* | 64 | 18/49 (36.7) | 84 | 21/47 (44.7) | 0.85 (0.61-1.17) | 0.322 |  |
| *Unknown* | 5 |  | 0 |  |  |  |  |
| **Drug-related AEs (any grade) other than SAE and AESI** | 16 | 11 (7.9) | 26 | 17 (12.5) | 0.65 (0.35-1.21) | 0.175 |  |
| ***Vaccination status*** |  |  |  |  |  |  | 0.096 |
| *0 injection* | 11 | 6/87 (6.9) | 11 | 8/88 (9.1) | 0.96 (0.42-2.22) | 0.929 |  |
| ≥*1 injection* | 4 | 4/49 (8.2) | 15 | 9/47 (19.2) | 0.30 (0.1-0.89) | 0.031 |  |
| *Unknown* | 1 |  | 0 |  |  |  |  |
| **Grade 3-4 AEs other than SAE and AESI** | 31 | 12 (8.6) | 37 | 16 (11.8) | 0.73 (0.45-1.19) | 0.206 |  |
| ***Vaccination status*** |  |  |  |  |  |  | 0.289 |
| *0 injection* | 26 | 7/87 (8.0) | 26 | 11/88 (12.5) | 0.96 (0.56-1.66) | 0.891 |  |
| ≥*1 injection* | 5 | 5/49 (10.2) | 11 | 5/47 (10.6) | 0.51 (0.18-1.46) | 0.207 |  |
| *Unknown* | 0 |  | 0 |  |  |  |  |
| **Disease related events (DREs)** | 83 | 83 (59.7) | 79 | 79 (58.1) | 0.99 (0.73-1.35) | 0.963 |  |
| ***Vaccination status*** |  |  |  |  |  |  | 0.609 |
| *0 injection* | 47 | 30/87 (34.5) | 48 | 27/88 (30.7) | 0.94 (0.63-1.41) | 0.773 |  |
| ≥*1 injection* | 31 | 15/49 (30.6) | 31 | 17/47 (36.2) | 1.11 (0.68-1.83) | 0.672 |  |
| *Unknown* | 5 |  | 0 |  |  |  |  |

*Cumulative incidence was compared between groups using Poisson regression analysis that accounted for all events and different follow-up durations for each participant.

**Table S5 continued:** Safety: Serious adverse events according to vaccination status

|  | **Non-Vaccinated** | | | **Vaccinated** | | |
| --- | --- | --- | --- | --- | --- | --- |
|  | **Placebo** | **Baricitinib** | **Total** | **Placebo** | **Baricitinib** | **Total** |
| **Respiratory, thoracic and mediastinal disorders** | **39** | **26** | **65** | **13** | **24** | **37** |
| *Acute respiratory distress syndrome* | *16* | *13* | *29* | *5* | *8* | *13* |
| *Pulmonary embolism* | *9* | *4* | *13* | *4* | *3* | *7* |
| *Respiratory failure* | *3* | *6* | *9* | *4* | *7* | *11* |
| *Pneumothorax* | *5* | *2* | *7* | *0* | *0* | *0* |
| *Hypoxia* | *2* | *1* | *3* | *0* | *0* | *0* |
| *Respiratory distress* | *2* | *0* | *2* | *0* | *0* | *0* |
| *Acute respiratory failure* | *0* | *0* | *0* | *0* | *1* | *1* |
| *Haemoptysis* | *0* | *0* | *0* | *0* | *1* | *1* |
| *Obstructive airways disorder* | *0* | *0* | *0* | *0* | *1* | *1* |
| *Pleural effusion* | *0* | *0* | *0* | *0* | *1* | *1* |
| *Pleuritic pain* | *0* | *0* | *0* | *0* | *1* | *1* |
| *Pneumonitis* | *1* | *0* | *1* | *0* | *0* | *0* |
| *Pulmonary hypertension* | *1* | *0* | *1* | *0* | *1* | *1* |
| **Infections and infestations** | **32** | **32** | **64** | **22** | **29** | **51** |
| *Pneumonia* | *15* | *17* | *32* | *10* | *9* | *19* |
| *Pneumonia bacterial* | *4* | *5* | *9* | *4* | *5* | *9* |
| *Septic shock* | *3* | *3* | *6* | *1* | *5* | *6* |
| *Bacteraemia* | *2* | *2* | *4* | *4* | *2* | *6* |
| *Bronchopulmonary aspergillosis* | *3* | *0* | *3* | *0* | *0* | *0* |
| *COVID-19 pneumonia* | *0* | *0* | *0* | *0* | *2* | *2* |
| *Sepsis* | *1* | *0* | *1* | *1* | *1* | *2* |
| *Cytomegalovirus infection reactivation* | *0* | *0* | *0* | *0* | *2* | *2* |
| *Fungaemia* | *0* | *0* | *0* | *0* | *2* | *2* |
| *Superinfection bacterial* | *1* | *1* | *2* | *0* | *0* | *0* |
| *Bacterial infection* | *0* | *1* | *1* | *0* | *0* | *0* |
| *Candida pneumonia* | *0* | *0* | *0* | *0* | *0* | *0* |
| *Device related bacteraemia* | *0* | *1* | *1* | *0* | *0* | *0* |
| *Disseminated aspergillosis* | *1* | *0* | *1* | *0* | *0* | *0* |
| *Hepatitis B reactivation* | *0* | *0* | *0* | *1* | *0* | *1* |
| *Herpes simplex* | *0* | *0* | *0* | *0* | *1* | *1* |
| *Infectious pleural effusion* | *1* | *0* | *1* | *0* | *0* | *0* |
| *Mediastinitis* | *0* | *1* | *1* | *0* | *0* | *0* |
| *Prostatitis Escherichia coli* | *0* | *0* | *0* | *1* | *0* | *1* |
| *Septic arthritis staphylococcal* | *1* | *0* | *1* | *0* | *0* | *0* |
| *Staphylococcal bacteraemia* | *0* | *1* | *1* | *0* | *0* | *0* |
| **Investigations** | **7** | **4** | **11** | **2** | **1** | **3** |
| *Transaminases increased* | *6* | *3* | *9* | *2* | *1* | *3* |
| *Blood bilirubin increased* | *1* | *0* | *1* | *0* | *0* | *0* |
| *C-reactive protein increased* | *0* | *1* | *1* | *0* | *0* | *0* |
| **Renal and urinary disorders** | **7** | **4** | **11** | **6** | **5** | **11** |
| *Acute renal failure* | *2* | *3* | *5* | *2* | *3* | *5* |
| *Acute kidney injury* | *3* | *1* | *4* | *3* | *1* | *4* |
| *Renal failure* | *2* | *0* | *2* | *0* | *1* | *1* |
| *Chronic kidney disease* | *0* | *0* | *0* | *1* | *0* | *1* |
| **General disorders and administration site conditions** | **2** | **3** | **5** | **3** | **5** | **8** |
| *Multiple organ dysfunction syndrome* | *2* | *3* | *5* | 3 | 3 | 6 |
| *Chest pain* | *0* | *0* | *0* | 0 | 1 | 1 |
| *Death* | *0* | *0* | *0* | 0 | 1 | 1 |
| **Blood and lymphatic system disorders** | **2** | **0** | **2** | **2** | **0** | **2** |
| *Anaemia* | *1* | *0* | *1* | *1* | *0* | *1* |
| *Bicytopenia* | *1* | *0* | *1* | *0* | *0* | *0* |
| *Thrombocytopenia* | *0* | *0* | *0* | *1* | *0* | *1* |
| **Cardiac disorders** | **4** | **2** | **6** | **1** | **3** | **4** |
| *Cardiac disorder* | *2* | *2* | *4* | *0* | *0* | *0* |
| *Cardiac failure* | *0* | *0* | *0* | *1* | *1* | *2* |
| *Acute coronary syndrome* | *1* | *0* | *1* | *0* | *0* | *0* |
| *Acute myocardial infarction* | *1* | *0* | *1* | *0* | *0* | *0* |
| *Bradycardia* | *0* | *0* | *0* | *0* | *1* | *1* |
| *Myocarditis / pericarditis* | *0* | *0* | *0* | *0* | *1* | *1* |
| **Nervous system disorders** | **3** | **0** | **3** | **0** | **1** | **1** |
| *Coma* | *0* | *0* | *0* | *0* | *1* | *1* |
| *Facial paralysis* | *1* | *0* | *1* | *0* | *0* | *0* |
| *Haemorrhage intracranial* | *1* | *0* | *1* | *0* | *0* | *0* |
| *Subarachnoid haemorrhage* | *1* | *0* | *1* | *0* | *0* | *0* |
| **Injury, poisoning and procedural complications** | **1** | **0** | **1** | **0** | **0** | **0** |
| *Hand fracture* | *1* | *0* | *1* | *0* | *0* | *0* |
| **Gastrointestinal disorders** | **2** | **0** | **2** | **1** | **0** | **1** |
| *Ileus* | *0* | *0* | *0* | *1* | *0* | *1* |
| *Intestinal ischaemia* | *1* | *0* | *1* | *0* | *0* | *0* |
| *Rectal haemorrhage* | *1* | *0* | *1* | *0* | *0* | *0* |
| **Vascular disorders** | **3** | **2** | **5** | **1** | **2** | **3** |
| *Deep vein thrombosis* | *2* | *1* | *3* | *1* | *1* | *2* |
| *Circulatory collapse* | *0* | *0* | *0* | *0* | *1* | *1* |
| *Hypotension* | *0* | *1* | *1* | *0* | *0* | *0* |
| *Peripheral artery thrombosis* | *1* | *0* | *1* | *0* | *0* | *0* |
| **Congenital, familial and genetic disorders** | **1** | **0** | **1** | **0** | **0** | **0** |
| *Atrial septal defect* | *1* | *0* | *1* | *0* | *0* | *0* |
| **Musculoskeletal and connective tissue disorders** | **0** | **0** | **0** | **1** | **0** | **1** |
| *Spondylolisthesis* | *0* | *0* | *0* | *1* | *0* | *1* |
| **Total** | **103** | **73** | **176** | **52** | **70** | **122** |

**Table S6:** Baseline demographics and clinical characteristics by prior vaccination for COVID-19 status

|  | **Non-vaccinated**  **(n=175)** | **Vaccinated**  **(n=96)** | **P-value**** |
| --- | --- | --- | --- |
| **Age (years), median (IQR)** | 56 (46-65) | 67 (57-74) | **<.0001** |
| n/N (%) |  |  |  |
| < 60 | 103/175 (58.9) | 30/96 (31.3) |  |
| ≥ 60 | 72/175 (41.1) | 66/96 (68.8) |  |
| **Gender, n/N (%)** |  |  | 0.5489 |
| Male | 132/175 (75.4) | 76/96 (79.2) |  |
| Female | 43/175 (24.6) | 20/96 (20.8) |  |
| **Country, n/N (%)** |  |  | **0.0187** |
| Austria | 6/175 (3.4) | 0/96 (0.0) |  |
| Belgium | 4/175 (2.3) | 4/96 (4.2) |  |
| France | 60/175 (34.3) | 29/96 (30.2) |  |
| Ireland | 5/175 (2.9) | 3/96 (3.1) |  |
| Italy | 17/175 (9.7) | 7/96 (7.3) |  |
| Luxembourg | 1/175 (0.6) | 0/96 (0.0) |  |
| Norway | 78/175 (44.6) | 42/96 (43.8) |  |
| Portugal | 2/175 (1.1) | 1/96 (1.0) |  |
| Spain | 2/175 (1.1) | 10/96 (10.4) |  |
| **Comorbidities, n/N (%)** |  |  |  |
| Obesity (BMI≥30 kg/m^2^) | 63/166 (38.0) | 35/88 (39.8) | 0.7879 |
| Diabetes | 29/174 (16.7) | 32/96 (33.3) | **0.0028** |
| Hypertension | 34/174 (19.5) | 50/96 (52.1) | **<.0001** |
| Chronic obstructive pulmonary disease | 5/174 (2.9) | 8/96 (8.3) | 0.0708 |
| Chronic cardiac disease | 19/174 (10.9) | 32/96 (33.3) | **<.0001** |
| Chronic kidney disease | 3/174 (1.7) | 8/96 (8.3) | **0.0191** |
| Chronic liver disease | 2/174 (1.2) | 3/96 (3.1) | 0.5824 |
| Cancer | 4/174 (2.3) | 6/96 (6.3) | 0.2780 |
| Autoimmune disease | 7/174 (4.0) | 5/96 (5.2) | 0.8453 |
| Immunodeficiency | 1/174 (0.6) | 3/96 (3.1) | 0.1289 |
| **Any comorbidities, n/N (%)** | 114/175 (65.1) | 83/96 (86.5) | **0.0002** |
| ***Days from symptoms onset to randomization*, n/N (%)** |  |  | 0.1430 |
| ≤7 | 43/175 (24.6) | 31/96 (32.3) |  |
| 8-13 | 125/175 (71.4) | 58/96 (60.4) |  |
| ≥ 14 | 7/175 (4.0) | 7/96 (7.3) |  |
| **WHO disease progression score, n/N (%)** |  |  | 0.1032 |
| 6 (severe disease) | 145/175 (82.9) | 87/96 (90.6) |  |
| 7-9 (critical disease) | 30/175 (17.1) | 9/96 (9.4) |  |
| **Concomitant medications, n/N (%)** |  |  |  |
| Remdesivir | 2/175 (1.1) | 6/96 (6.3) | **0.0251** |
| Systemic corticosteroids | 169/175 (96.6) | 88/96 (91.7) | 0.0917 |
| Anticoagulants | 161/175 (92.0) | 86/96 (89.6) | 0.5095 |
| **Biochemistry, median (IQR)** |  |  |  |
| Lymphocyte count (10^9^ cells/L) |  |  | 0.7711 |
| *N* | 173 | 91 |  |
| *Median (IQR)* | 0.8 (0.6-1.1) | 0.8 (0.5-1.1) |  |
| Neutrophil count (109 cells/L) |  |  | **0.0573** |
| *N* | 173 | 91 |  |
| *Median (IQR)* | 5.8 (4.1-7.9) | 6.5 (4.3-9.4) |  |
| C-reactive protein (mg/L) |  |  | **0.0544** |
| *N* | 170 | 94 |  |
| *Median (IQR)* | 83 (39-138) | 93 (59-143) |  |
| Ferritin (ng/mL) |  |  | **0.0014** |
| *N* | 150 | 74 |  |
| *Median (IQR)* | 1283 (750-1960) | 833 (468-1555) |  |
| Lactate dehydrogenase (U/L) |  |  | **0.0004** |
| *N* | 154 | 77 |  |
| *Median (IQR)* | 432 (338-555) | 371 (266-468) |  |
| Procalcitonin (ng/mL) |  |  | **0.0214** |
| *N* | 132 | 55 |  |
| *Median (IQR)* | 0.1 (0.1-0.3) | 0.2 (0.1-0.4) |  |
| D-dimer (μg/L FEU) |  |  | 0.9874 |
| *N* | 162 | 79 |  |
| *Median (IQR)* | 900 (600-1480) | 900 (590-1700) |  |
| **SARS-CoV2 serostatus, n/N (%)** |  |  | **<.0001** |
| Negative | 43/75 (57.3) | 3/49 (6.1) |  |
| Positive | 32/75 (42.7) | 46/49 (93.9) |  |
| **Nasopharyngeal viral load, (Log copies/10000 cells)** **median (IQR)*** |  |  | **0.0324** |
| *N* | 79 | 45 |  |
| *Median (IQR)* | 3.0 (1.6-4.3) | 4.0 (2.6-5.0) |  |
| Viral load value <LOQ | 14/79 (17.7) | 4/45 (8.9) |  |

SARS-CoV2 serostatus and nasopharyngeal viral load were done in participants with biobank. SARS-CoV2 serostatus was based on the anti-RBD WT (BAU/mL) value with a cutoff of 10.

* Values below the limit of quantification (LOQ) were replaced by the LOQ value (LOQ=1)

**P-values were obtained from Mann-Whitney test for numeric variables and from Fisher’s exact test for categorical variables. The p-values are purely descriptive and are included to give indications of non-random differences between the groups.
